# Metabolic Engineering of *Saccharomyces cerevisiae* for Heterologous Carnosic Acid Production

**DOI:** 10.3389/fbioe.2022.916605

**Published:** 2022-06-02

**Authors:** Panpan Wei, Chuanbo Zhang, Xueke Bian, Wenyu Lu

**Affiliations:** ^1^ School of Chemical Engineering and Technology, Tianjin University, Tianjin, China; ^2^ Key Laboratory of Systems Bioengineering of the Ministry of Education, Tianjin University, Tianjin, China; ^3^ SynBio Research Platform, Collaborative Innovation Center of Chemical Science and Engineering, Tianjin, China

**Keywords:** carnosic acid, *Saccharomyces cerevisiae*, terpenoid, miltiradiene, synthetic biology

## Abstract

Carnosic acid (CA), a phenolic tricyclic diterpene, has many biological effects, including anti-inflammatory, anticancer, antiobesity, and antidiabetic activities. In this study, an efficient biosynthetic pathway was constructed to produce CA in *Saccharomyces cerevisiae*. First, the CA precursor miltiradiene was synthesized, after which the CA production strain was constructed by integrating the genes encoding cytochrome P450 enzymes (P450s) and cytochrome P450 reductase (CPR) SmCPR. The CA titer was further increased by the coexpression of CYP76AH1 and SmCPR ∼t28SpCytb5 fusion proteins and the overexpression of different catalases to detoxify the hydrogen peroxide (H_2_O_2_). Finally, engineering of the endoplasmic reticulum and cofactor supply increased the CA titer to 24.65 mg/L in shake flasks and 75.18 mg/L in 5 L fed-batch fermentation. This study demonstrates that the ability of engineered yeast cells to synthesize CA can be improved through metabolic engineering and synthetic biology strategies, providing a theoretical basis for microbial synthesis of other diterpenoids.

## Introduction

Diterpenoids have diverse structures and biological activities (Wang and Weller, 2006). Naturally occurring cyclic diterpenoids, such as tanshinones, paclitaxel, or platensimycin, possess antimicrobial and antitumor activities. Among them, paclitaxel has been extensively used as an anticancer drug (Vranová et al., 2013; Sultan et al., 2021). Ginkgo lactone can be used to treat cardiovascular disease as a natural platelet activation antagonist (Liu et al., 2018) and is also employed to treat Alzheimer’s disease (Sarkar et al., 2020). Carnosic acid (CA) is a phenolic tricyclic diterpene (Mena et al., 2016) that has attracted much interest for its pharmacological properties against obesity, neurodegenerative ailment, and cancer (Barni et al., 2012; Dickmann et al., 2012). At the same time, CA is extensively utilized in many other fields due to its safety, nontoxicity, and high-temperature resistance, including pharmaceuticals, cosmetics, food additives, and spices (Jordán et al., 2012; Ou et al., 2018).

**FIGURE 1:**
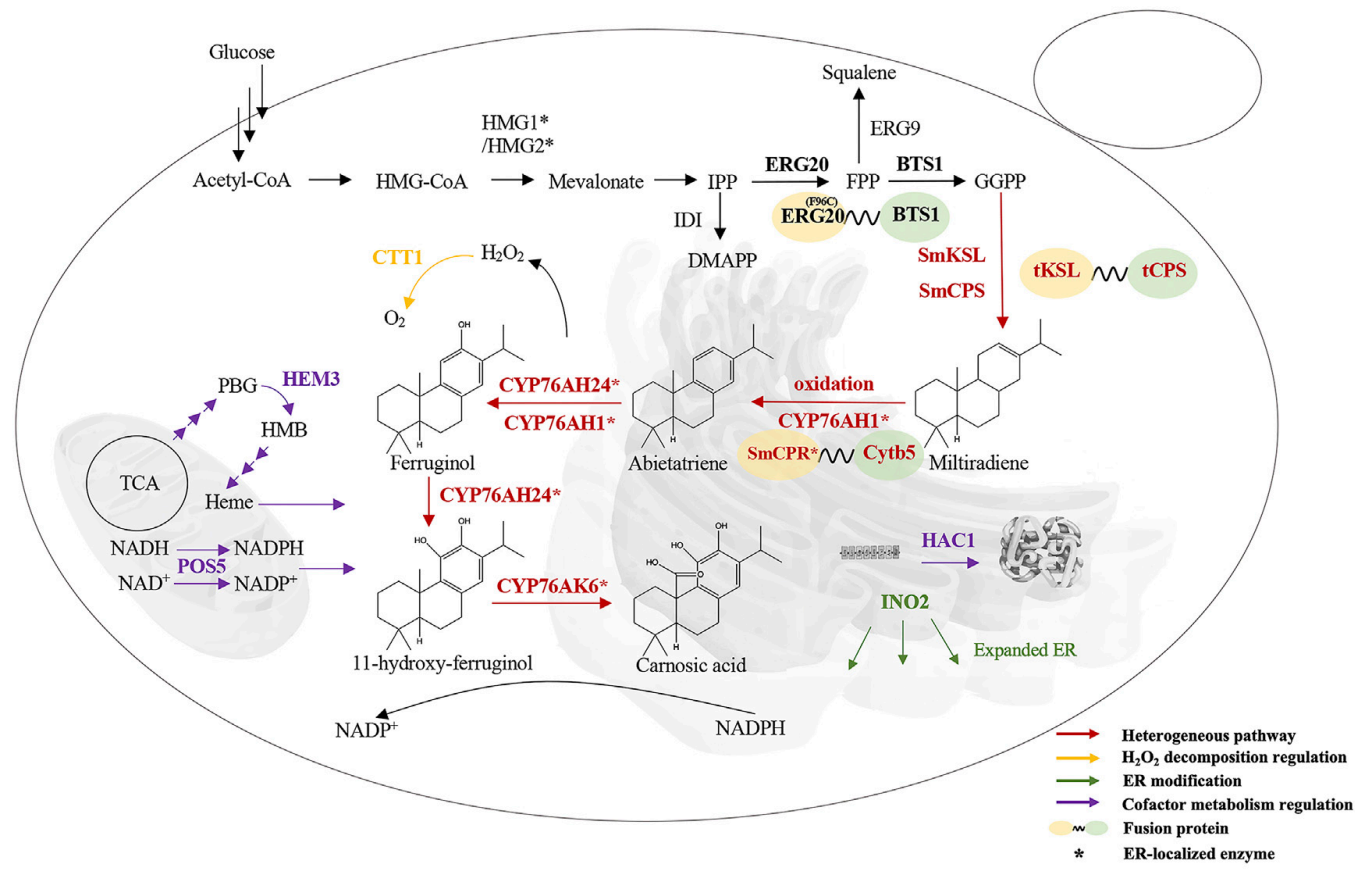
Overall strategy of CA production in *S. cerevisiae*. HMG1/HMG2, HMG-CoA reductase; IDI, isopentenyl pyrophosphate isomerase; ERG20, FPP synthase; ERG9, squalene synthetase; BTS1, GGPP synthase; SmCPS/SmKSL, miltiradiene synthase; CYP76AH24, 11-hydroxy-ferruginol synthase; CYP76AK6, CA synthase; CYP76AH1, ferruginol synthase; HMG-CoA, hydroxymethylglutaryl-CoA; SmCPR, cytochrome P450 reductase; Cytb5, cytochrome B5; CTT1, catalase; ER, endoplasmic reticulum; INO2, transcription factor that promotes phospholipid synthesis; HAC1, transcription factor that promotes protein folding; HEM3, heme synthase; TCA, tricarboxylic acid cycle; POS5, NADH kinase. Three arrows represent multistep reactions.

At present, phytoextraction is the primary source of commercial CA, but the content of terpenoids in the plant is low (Liu and Khosla, 2010; Weathers et al., 2011), and the phytoextraction approach is restricted by the supply of plant material (Pickens et al., 2011; Birtic et al., 2015). Plant terpenoids synthesis by microorganisms is an effective method to solve these problems. *Saccharomyces cerevisiae* has a clear genetic background and is among the most thoroughly studied eukaryotes. In addition, *S. cerevisiae* can be easily cultured, grows rapidly, and possesses a natural mevalonate acid (MVA) pathway, which makes it convenient for extensive industrial usage (Tholl, 2006). *S. cerevisiae* is widely used in the microbial synthesis of terpenoids (Paramasivan and Mutturi, 2017), such as the monoterpene alcohol citronellol (Jiang et al., 2021), the sesquiterpene artemisinic acid (Paddon et al., 2013), the diterpenoid paclitaxel precursor taxadiene (Nowrouzi et al., 2020), the triterpenoid ginsenoside compound K (Shi et al., 2021), and the tetraterpenoid carotenoids (Cataldo et al., 2020). CA production and metabolic pathway reconstruction were also accomplished in *S. cerevisiae*. The biosynthetic route of CA is shown in Figure 1. A copalyl diphosphate synthase (CPS) and a kaurene synthase-like (KSL) enzyme catalyze geranylgeranyl diphosphate (GGPP) into miltiradiene (Gao et al., 2009; Brueckner et al., 2014). Several academics have suggested that the conversion of miltiradiene into abietatriene is a spontaneous oxidation process (Ignea et al., 2016; Scheler et al., 2016). CYP76AH24 catalyzes the oxidation of the labdane skeleton at C-12 and C-11 to produce 11-hydroxy-ferruginol, which is then catalyzed by CYP76AK6 to produce CA in the final steps (Ignea et al., 2016). Furthermore, CYP76AH1 can reportedly directly oxidize miltiradiene and produce ferruginol (Guo et al., 2013). As an essential precursor of CA, the biosynthesis of miltiradiene is of great interest. Dai et al. obtained 365 mg/L miltiradiene by the fusion of SmKSL with SmCPS and ERG20 with BTS1, together with the optimization of the MVA pathway (Dai et al., 2012). Through further screening, fusion, and truncation of diterpene synthases and enhancement of the GGPP supply, the titer of miltiradiene reached approximately 3,500 mg/L (Hu et al., 2020). Ignea and colleagues first constructed an *S. cerevisiae* platform for CA production. They fused the FPP synthase mutant Erg20p^(F96C)^ with CPP synthase and expressed the *HEM3* gene, leading to 1 mg/L CA (Ignea et al., 2016). Then, they adjusted the linker length used for the fusion protein and balanced the co-expression of cytochrome P450 reductase (CPR), cytochrome P450 enzymes (P450s), and cytochrome b5 (Cytb5), leading to 18 mg/L CA (Ignea et al., 2017). In addition, Scheler et al. (2016) expressed GGPP synthase, miltiradiene synthase, CPS, ATR1, and the two P450s CYP76AH1 and CYP76AK8, obtaining 2.74 mg/L CA. However, the CA titer was still meager compared to miltiradiene, which indicates that the rate-limiting step is the conversion of miltiradiene by P450s.

Researchers have used many engineering strategies targeting P450s to improve terpenoid production. Zhu et al. (2018) boosted the yield of total triterpenoids 5.61-fold by screening CPRs from different plant sources. Our lab fused P450s with CPR and increased the enzyme activity 4.5-fold (Zhao et al., 2016). Our lab overexpressed the endoplasmic reticulum (ER) size regulatory factor *INO2* to boost P450s expression in ER. As a result, forskolin titer increased by 2.61 times in *S. cerevisiae* (Ju et al., 2022). These efforts are significant and helpful for CA pathway optimization. However, when exogenous pathways are introduced into *S. cerevisiae*, regulation of the rate-limiting step alone is not sufficient to maximize the synthesis of the target product.

In this study, CA was produced in *S. cerevisiae* by integrating a heterologous biosynthesis pathway, as shown in Figure 1. Further metabolic optimization mainly focused on P450s modules and the endogenous metabolic network boosted the CA titer to 24.65 mg/L in shake flasks and 75.18 mg/L in 5 L fed-batch fermentation.

## Materials and Methods

### Strains, Reagents, and Culture Media

For the development of all engineered strains, *S. cerevisiae* 3HP-F was utilized as the initial strain (Zhang et al., 2019). Jinkairui Biotechnology Co., Ltd. (Wuhan, China) codon optimized and synthesized the DNA fragments encoding SmCPS (copalyl diphosphate synthase) (GenBank: EU003997.1), SmKSL (kaurene synthase-like enzyme) (GenBank: EF635966.2), CYP76AH24 (11-hydroxy-ferruginol synthase) (GenBank: KT157044.1), CYP76AK6 (CA synthase) (GenBank: KT157045.1), GuCPR (cytochrome P450 reductase) (GenBank: QCZ35624.1) and AtCPR (GenBank: BT008426.1), and cloned them into the plasmid pUC57. GENEWIZ (Beijing, China) codon optimized and synthesized the DNA fragment encoding SmCPR (GenBank: CBX24555). Yeast transformants were screened on synthetic medium lacking specific amino acids. Standard culture was conducted in yeast extract peptone dextrose medium (YPD), which comprised 2% peptone, 1% yeast extract, and 2% glucose. GENEWIZ (Beijing, China) synthesized the primers. TIANGEN (Beijing, China) provided the mini plasmid extraction and DNA gel mini purification kits. *S. cerevisiae* W303-1a genomic DNA was used to amplify the promoter, terminator, optional marker, and other native sequences.

### Construction and Integration of Yeast Expression Cassettes

We used the lithium acetate method to transform *S. cerevisiae* as described previously (Gietz and Schiestl, 2007). The expression cassette containing the coding DNA sequence, the promoter and terminator was constructed using fusion polymerase chain reaction (PCR). The expression cassettes and the expression fragments are shown in Supplementary Table S1 and Supplementary Figure S1. The primers, coding DNA sequences, promoter and terminator sequences used in the construction of strains are shown in Supplementary Tables S2–S4.

### Cultivation and Fermentation of Yeast

A single colony was inoculated into a tube containing 3 ml of YPD medium and cultivated for 16–18 h at 220 rpm at 30°C. The culture was then inoculated into a shake flask with 30 ml of YPD medium to an initial OD_600_ of 0.05, then cultivated for 96 h under the same condition. All the experiments in conical flasks were done in triplicate. A conventional spectrophotometer (Oppler, 752 N, China) was used to determine the OD_600_.

### Extraction and Analysis of Metabolites

Miltiradiene, ferruginol, and CA were extracted using n-hexane from lysed cells and the supernatant of the fermentation broth. The fermentation broth was centrifuged at 10000 g for 10 min. The cell precipitate and supernatant were separated into two centrifuge tubes, after which n-hexane was added to both, and additional glass beads were added to the cell precipitate. Both tubes were then shaken vigorously in a vortex shaker for 40 min and centrifuged. The upper organic phases were aspirated with a syringe and combined. Ferruginol and CA standards were purchased from Solarbio (Beijing, China).

Miltiradiene was identified by gas chromatography-mass spectrometry. A Shimadzu GC-MS-TQ8030 apparatus with a GC column HP-5ms (Agilent Technologies, 30 m × 0.250 mm × 0.25 μm) was used to analyze the samples (1 μL). The temperature gradient was as follows: injection temperature, 250°C; 5 min at 150°C, ramp at 5°C/min to 250°C, then hold for 5 min. The spectra were scanned between 30 and 550 m/z with an ion source temperature of 260°C.

Ferruginol and CA were identified by liquid chromatography-mass spectrometry (LC-MS). High-performance liquid chromatography (HPLC) with an Elite P230II high-pressure pump system was used to quantify ferruginol and CA. A Grace Apollo C18 column (4.6 × 250 mm, 5 mm) was used for chromatographic separation. The detection ultraviolet is 230 nm. The LC-MS analysis was carried out using an Agilent Zorbax SB Aq column (100 mm × 2.1 mm × 3 μm) with Surveyor LC System (Thermo Finnigan, San Jose, CA, United States). Negative ionization was used to evaluate the samples. The elution conditions were as follows: the injection volume was 30 μL; the eluent was 40:60 water: acetonitrile; the column temperature was 30°C; the flow rate was 1 ml/min; scanning mode: first-level full scan. For compound identification, we compared the mass spectra and retention times with authentic standards.

A bioanalyzer (SBA-40C, Shandong Academy of Sciences, China) and an Aminex HPX-87H column (Bio-Rad, United States) were used to measure glucose and ethanol concentrations. The flow rate was 0.6 ml/min; the eluent was 5 mM H_2_SO_4_; the column temperature was 65°C.

### Compound Quantification and Statistical Analysis

The internal standard for miltiradiene quantitation was 1-eicosene. A calibration curve with an R^2^ coefficient of more than 0.99 (Supplementary Figure S6) was used to quantify the ferruginol and CA in LC analysis. The highest and lowest deviations from three different cultivations were represented by error bars. Univariate analysis (*t*-test) was used to assess the statistical significance (*p*-value).

### Fluorescence Measurement

A single colony was inoculated into a tube containing 3 ml of YPD medium and cultivated for 16–18 h at 220 rpm at 30°C. The culture was used to inoculate another tube with 3 ml of YPD medium to 0.2 initial OD_600_, then cultivated at 220 rpm and 30°C for 48 h for fluorescence measurements using Infinite 200 PRO Multimode Microplate Reader (TECAN, Switzerland). All the experiments were done in triplicate.

### Quantitative PCR for Gene Copy Number Detection

The number of integrated heterologous expression cassettes was determined using absolute qPCR with *Arg4* as an internal reference gene (Abad et al., 2010). We used the primers listed in Supplementary Table S2 to quantify the open reading frames of CYP76AH24, CYP76AK6, CYP76AH1, SmCPR, and ARG4 genes to generate standard curves. Genomic DNA was extracted from distinct colonies and three repeated qPCR assays were conducted using the LightCycler 480 System with TaqMan probe qPCR TB Green Premix Ex Tap II (Vazyme, China). Detection based on the TaqMan probe was described by Zhang et al. (2014).

### Fed-Batch Cultivation

The preserved engineered *S. cerevisiae* strains were streaked onto a YPD plate and activated to obtain a single colony. This single colony was cultured in a test tube containing 3 ml of YPD at 220 rpm and 30°C for 16–18 h, and the resulting seed culture was transferred to 200 ml of YPD in a shake flask and cultured for 16–18 h under the same condition. The secondary seed culture was inoculated 2 L of YPD medium in a 5 L fermenter at a volume ratio of 1:10. The automatic addition of 2.5 M sulfuric acid and 5 M ammonia kept the pH constant at 5.5. The airflow was 2 vvm, the fermentation temperature was 30°C, and the rotation speed was varied between 200 and 600 rpm to maintain the dissolved oxygen (DO) above 35% of the atmospheric value.

The conditions for two-stage fed-batch fermentation were the same as the batch fermentation. When the initial glucose was completely spent, feeding was initiated with a solution containing 500 g/L glucose, 5.12 g/L MgSO_4_·7H_2_O, 0.28 g/L Na_2_SO_4_, 3.5 g/L K_2_SO_4_, 9 g/L KH_2_PO_4_, 0.6 g/L uracil, 0.5 g/L adenine, 1.2 g/L lysine, 12 ml vitamin solution and 10 ml trace element solution. As previously reported, the vitamin and trace element solution was prepared (Zhou et al., 2010). When glucose consumption was complete and the ethanol produced in the process of metabolism was consumed, feeding with ethanol (95%, V/V) was initiated and the ethanol concentration in the fermenter was kept at 1–6 g/L in addition to 350 ml of feeding solution components other than glucose was added. The highest and lowest deviations from three different cultivations were represented by error bars.

## Results

### Reconstructing the Miltiradiene Biosynthesis Pathway in *S. cerevisiae*

**FIGURE 2:**
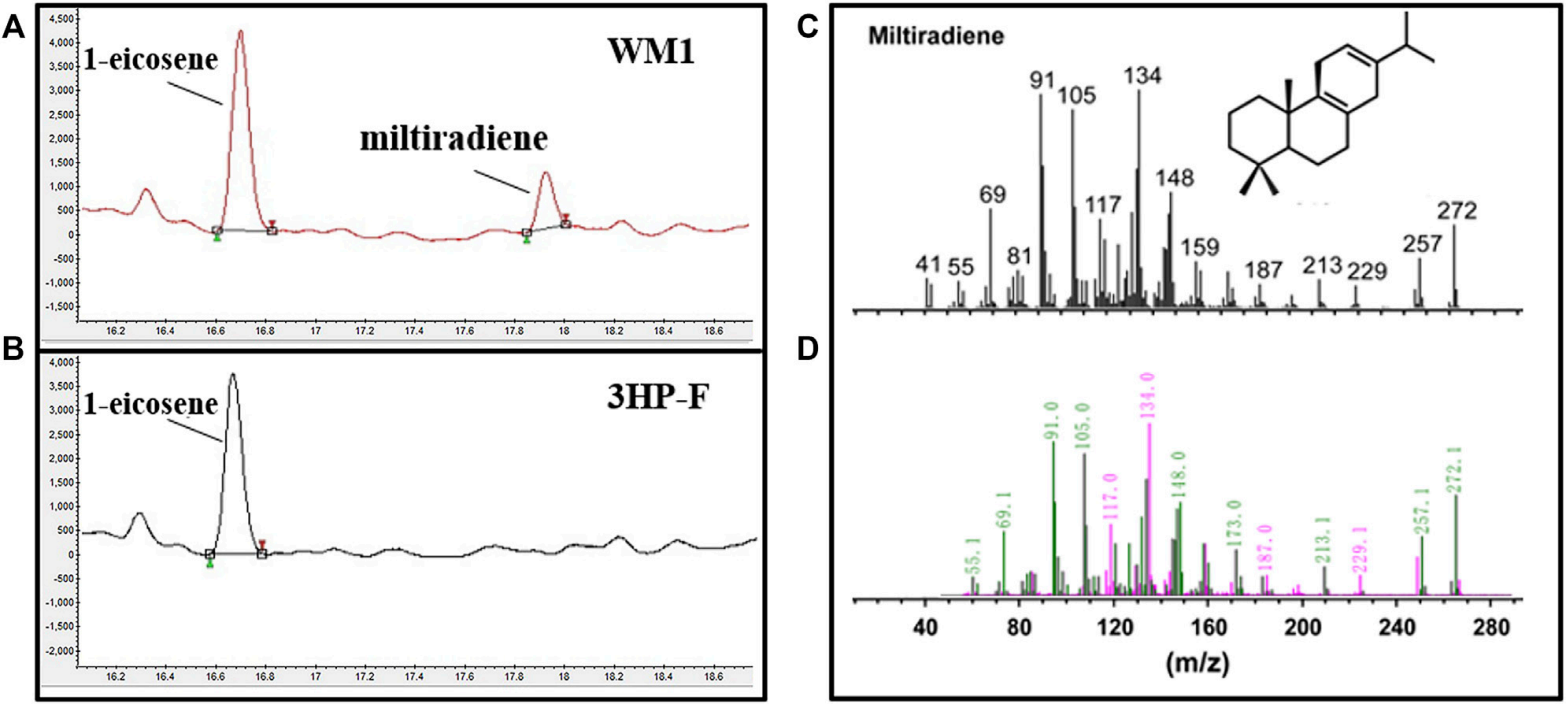
Miltiradiene production in *S. cerevisiae*. **(A)** Chromatogram of miltiradiene produced by strain WM1; **(B)** Chromatogram of the original strain 3HP-F; **(C)** GC–MS spectra of miltiradiene published in the literature (Hu et al., 2020); **(D)** GC–MS spectra of the chromatographic peak at RT = 17.82 min.

The codon-optimized miltiradiene synthesis genes from *Salvia miltiorrhiza* (SmCPS and SmKSL) were integrated into *S. cerevisiae* 3HP-F to form the WM1 strain. The strong promoters *P*_*PGK1*_ and *P*_*TDH3*_ were selected to express these two genes, respectively. Based on published mass spectra, the new peak (retention time (RT) = 17.82 min) was identified as miltiradiene (Hu et al., 2020) (Figure 2). We compared the peak areas of the internal standard 1-eicosene and miltiradiene to quantify a titer of 0.18 mg/L for miltiradiene after 4 days of shake flask culture.

**FIGURE 3:**
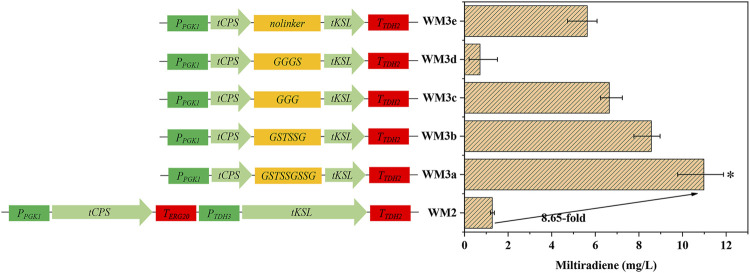
Protein fusion and linker selection. WM2: Coexpression of *tSmCPS* and *tSmKSL*. WMa–e: Fusion of tSmCPS and tSmKSL using five flexible linkers: GSTSSGSSG, GSTSSG, GGG, GGGS, no linker. *: The product titer of strain WM3a was significantly higher than that of WM3b. *p < 0.05; **p < 0.01; ***p < 0.001. Histograms illustrating the production of corresponding strains. Experiments were performed in triplicate.

Plant diterpenes are synthesized in plastids, and diterpene synthases usually contain an N-terminal plastidic transit peptide (Bohlmann et al., 1998). However, the transit peptide was reported to hinder the heterologous expression of diterpene synthases (Hu et al., 2020). Here, we identified the transit peptide of miltiradiene synthases using the ChloroP online tool (https://www.cbs.dtu.dk/services/ChloroP/) to construct the truncated variants t*SmCPS* and t*SmKSL*. Supplementary Table S3 shows the plastidic transit peptide coding sequences. The strong promoters *P*_*PGK1*_ and *P*_*TDH3*_ were selected to co-express t*SmCPS* and t*SmKSL* from the *ura3* locus of the strain 3HP-F to form WM2, which produced 1.27 mg/L miltiradiene, representing a 7.06-fold increase over the original strain (Figure 3). Jiang et al. (2017) removed the plastidic transit peptide from the N-terminus of geraniol synthase and fused a red fluorescent protein to its C-terminus to characterize the expression level of the correctly folded protein using fluorescence measurements. To investigate the expression of tSmCPS and tSmKSL, we fused the red fluorescent protein mCherry to the C-terminus of SmCPS, tSmCPS, SmKSL, and tSmKSL and expressed these four fusion proteins in *S. cerevisiae*. The relative fluorescence (RFU) of tSmCPS and tSmKSL was respectively 2.1 and 2.5 times higher than that of SmCPS and SmKSL, indicating that the expression of both enzymes was enhanced (Supplementary Figure S2).

Fusion proteins can boost the effective local concentration of substrates and enzymes, improving the product yield. Linker length has a crucial effect on the function of fusion proteins (Haga et al., 2013). For example, extremely long linkers make the fusion proteins prone to degradation and affect the product yield, while extremely short linkers affect the spatial conformation of the protein, which causes it to fail to play its original catalytic role. We fused tSmCPS and tSmKSL using five flexible linkers to obtain strains WM3a-e (Figure 3). The GSTSSGSSG linker had the best effect, and the titer of miltiradiene reached 10.98 mg/L, 8.65 times higher than strain WM2, co-expressing tSmCPS and tSmKSL (Supplementary Figure S3). Overexpression of the *BTS1*-GGGS-*ERG20*, a fusion of two critical enzymes of the MVA pathway, can considerably increase miltiradiene production (Zhou et al., 2012). In addition the *ERG20* ^(F96C)^ mutant can considerably enhance GGPP accumulation while having no apparent effect on FPP production (Ignea et al., 2015). Accordingly, *BTS1*-GGGS-*ERG20* ^F96C^p was overexpressed to create the WM4 strain, which reached a miltiradiene titer of 172.77 mg/L (Supplementary Figure S3).

### Selection of Cytochrome P450 Reductases for Cytochrome P450 Enzymes (P450s) to Produce Carnosic Acid

P450s are frequently utilized in the synthesis of drugs, vitamins, and spices (Ignea et al., 2015). However, few plant P450s exhibited high activity, and have been optimized using protein engineering, metabolic engineering, redox chaperone engineering, and substrate engineering approaches to increase terpene production (Xiao et al., 2019). Thus, choosing an appropriate functional cytochrome P450 reductase (CPR) is essential for maximizing the redox coupling efficiency.

**FIGURE 4:**
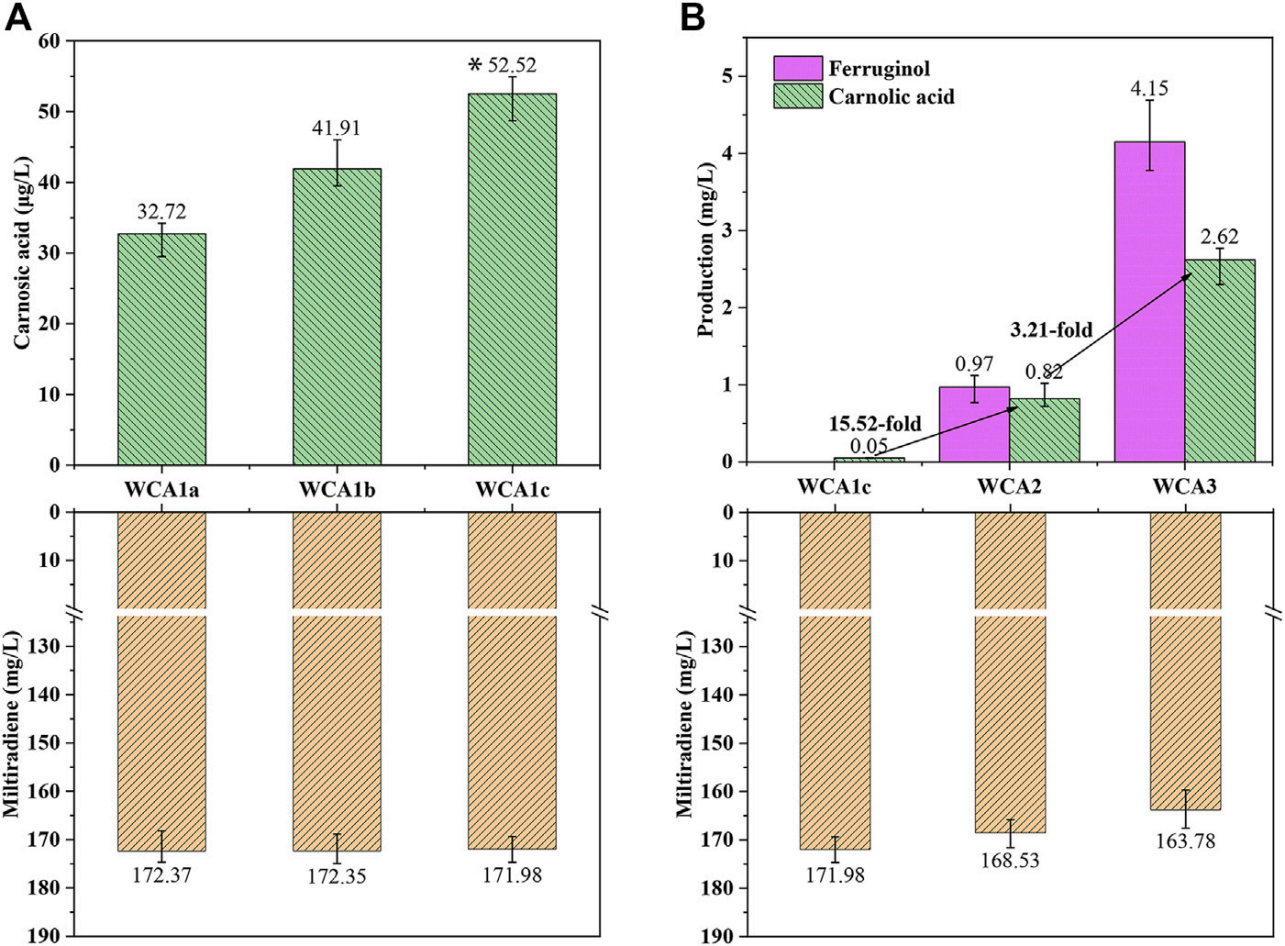
CA production in *S. cerevisiae* increased in a stepwise manner. **(A)** Production of CA and miltiradiene by strains WCA1a–c. *: The CA titer of strain WCA1c is significantly higher than that of WCA1b. *p < 0.05; **p < 0.01; ***p < 0.001 **(B)** Production of miltiradiene, ferruginol and CA by strain WCA2-3. The data are the averages of three separate experiments.

Three distinct CPR encoding genes were chosen for co-expression with P450s *CYP76AH24* and *CYP76AK6* (from *S. miltiorrhiza*) in WM4, resulting in strains WCA1a (*AtCPR* from *Arabidopsis thaliana*), WCA1b (*GuCPR* from *Glycyrrhiza uralensis*), and WCA1c (*SmCPR* from *S. miltiorrhiza*). The processed samples were analyzed using LC–MS after 4 days of culture. As shown in Supplementary Figure S4, CA and ferruginol were identified as new peaks by LC–MS analysis. WCA1c containing *SmCPR* produced 52.5 μg/L of CA, 70% more than WCA1a containing *AtCPR* and 30% more than strain WCA1b containing *GuCPR* (Figure 4A). A high coupling efficiency and electron transfer compatibility were reported in the homologous CYP-CPR reconstituted system, which boosted the monooxygenase activity (Dietrich et al., 2005; Jensen and Møller, 2010), and was consistent with our findings.

The conversion rate of the precursor miltiradiene was low, and may be attributed to the low efficiency of spontaneous oxidation of miltiradiene into abietatriene. By contrast, *CYP76AH1* can reportedly directly oxidize miltiradiene to produce ferruginol (Guo et al., 2013). To further improve the CA titer, we overexpressed *CYP76AH1* to develop the WCA2 strain. The ferruginol and CA titers reached 0.97 and 0.81 mg/L, indicating that *CYP76AH* expression could increase the conversion of miltiradiene into ferruginol (Figure 4B).

Cytochrome B5 (Cytb5) acts as an electron transporter in many biological oxidation reactions (Zhang et al., 2007). It appears to act as a specific electron donor when involved in catalysis together with NADPH-cytochrome B5 reductase or NADPH-CPR (Gilep et al., 2001). Therefore, we overexpressed Cytb5 from *S. pomifera* to develop the WCA3 strain with a CA titer of 2.62 mg/L, which was 3.21 times higher than in WCA2. Furthermore, the ferruginol titer also increased 4.27 times, reaching 4.15 mg/L (Figure 4B).

### Optimization of Carnosic Acid Production by the Fusion of Cytochrome P450 Monooxygenase and Cytochrome P450 Reductases

**FIGURE 5:**
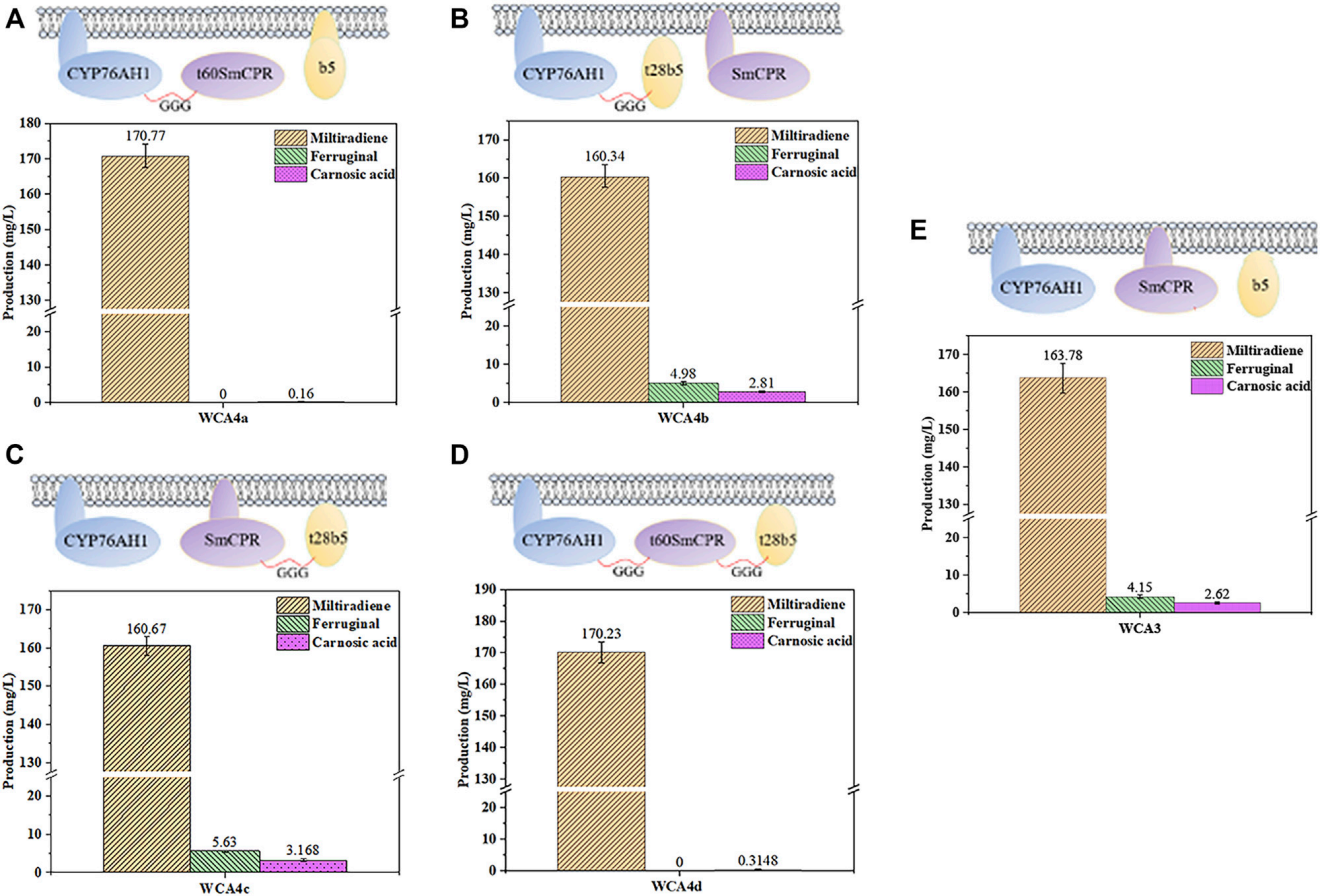
Influence of different fusion strategies on miltiradiene, ferruginol and CA production. **(A)** WCA4a: CYP76AH1 ∼ t60SmCPR, SpCytb5; **(B)** WCA4b: SmCPR, CYP76AH1 ∼ t28SpCytb5; **(C)** WCA4c: SmCPR ∼ t28SpCytb5, CYP76AH1; **(D)** WCA4d: CYP76AH1 ∼ t60SmCPR ∼ t28SpCytb5. **(E)** WCA3: CYP76AH1, SmCPR and SpCytb5 in the natural state. The data are the averages of three separate experiments.

The fusion of CPR with a compatible P450 enzyme could reportedly result in evolutionary advantages in terms of catalytic efficiency (McLean et al., 2007), and a fusion of the P450s CYP3A4, NADPH-CPR, and Cytb5 showed high activity (Inui et al., 2007). Therefore, we fused CYP76AH1, SmCPR, and SpCytb5 to explore the optimal fusion strategy for CA production. In our previous study, protopanaxadiol synthase and a truncated variant of ATR1 without its N-terminal transmembrane region were used to construct a fusion protein with higher activity (Zhao et al., 2016). Here, we used the TMHMM server to predict the transmembrane regions of SmCPR and SpCytb5, as shown in Supplementary Table S3. First, the truncated SmCPR was fused with CYP76AH1 and co-expressed with native SpCytb5 to construct the WCA4a strain. However, the results showed a sharp decline in CA production relative to the WCA3 strain (Figures 5A,E). We then fused the truncated SpCytb5 with CYP76AH1 and SmCPR, respectively, and then co-expressed it with native SmCPR or CYP76AH1, resulting in the strains WCA4b and WCA4c. The results showed that the CA titer of WCA4b and WCA4c was improved compared with WCA3 (Figures 5B,C,E), whereby the product titer of the best strain WCA4c reached 3.17 mg/L (Figure 5C), indicating that the fusion of truncated SpCytb5 and SmCPR improved the electron transport efficiency. Finally, we fused the truncated SmCPR, truncated SpCytb5, and CYP76AH1 to construct the WCA4d strain. However, its CA titer was unsatisfactory (Figure 5D). According to these results, we found that after excising the transmembrane region of SmCPR protein, the CA titer was significantly reduced regardless of the fusion method (Figure 5). In addition, we detected carnosol, a spontaneous oxidation product of CA, in WCA4c, which is in line with earlier research (Scheler et al., 2016). Because the product titer of strain WCA4c showed the largest increase, we chose the coexpression of SmCPR∼t28SpCytb5 fusion protein and CYP76AH1 for further experiments.

To further improve CA production, we chose a multicopy site to integrate the expression cassettes encoding the CA synthesis pathway. First, WCA4c was used as the chassis strain, and the *SmCPR∼t28SpCytb5* fusion gene and *CYP76AH1* were co-expressed from the δ site to obtain the WCA5 strain with CA and ferruginol titers of 4.30 and 12.74 mg/L, respectively. Then, we integrated the *CYP76AH24* and *CYP76AK6* genes into the ribosomal DNA site, resulting in the WCA6 strain with the highest CA titer of 6.20 mg/L. The gene copy number of strain WCA6 is shown in Supplementary Figure S5.

### Expression of Catalase-Related Genes in *S. cerevisiae*

The normal aerobic metabolism of *S. cerevisiae* is accompanied by the generation of reactive oxygen species (Temple et al., 2005). In the heterologous synthesis of terpene products in microorganisms, P450s and their reductases are sometimes poorly coupled, resulting in the generation of reactive oxygen species (Paddon et al., 2013). In the CA production strain, we expressed three heterologous P450s, which may have caused excessive accumulation of reactive oxygen species. H_2_O_2_ is accumulated due to ATP synthesis and spontaneous or enzymatic hydrolysis of O_2_ during respiratory metabolism, which can cause damage to the cell (Temple et al., 2005). Catalase is a primary H_2_O_2_ scavenging enzyme essential for preserving intracellular homeostasis of reactive oxygen species. In *S. cerevisiae*, the ScCTA1 catalase is localized to peroxisomes and mitochondria, where it degrades H_2_O_2_ produced during aerobic respiration and β-oxidation (Dzanaeva et al., 2020). The second ScCTT1 catalase is localized in the cytoplasm and responds to oxidative stress caused by H_2_O_2_ accumulation (Martins et al., 2019).

**FIGURE 6:**
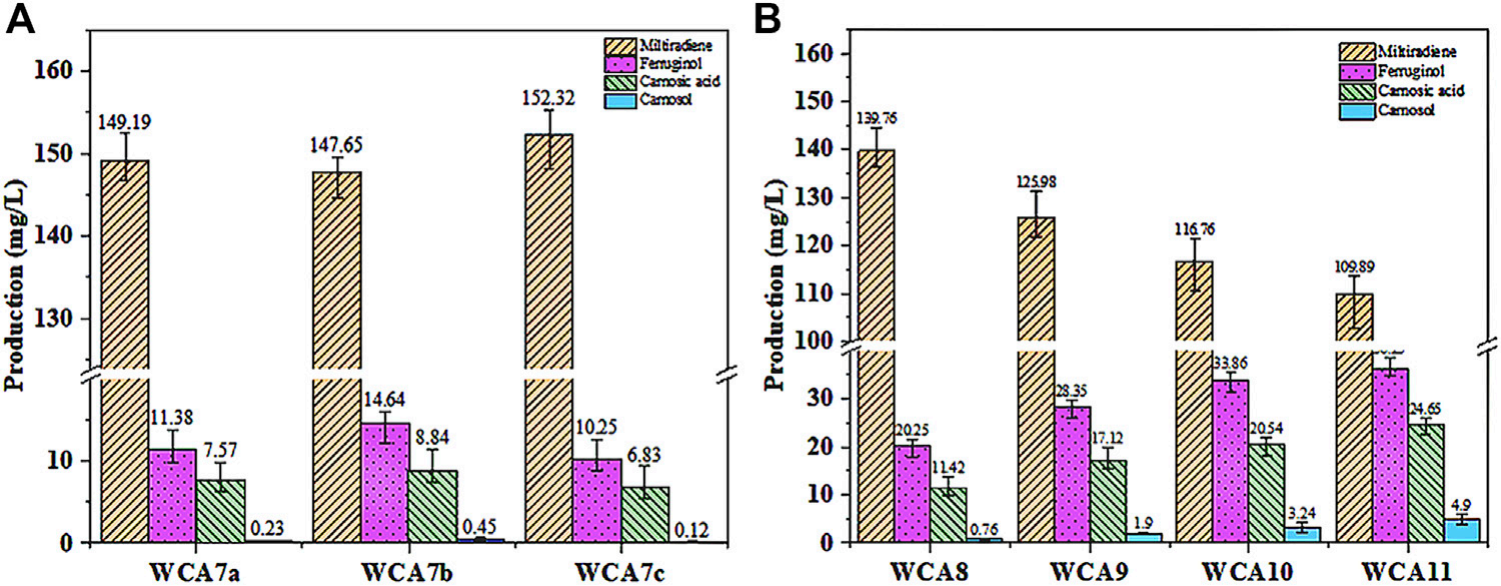
CA production in *S. cerevisiae* increased in a stepwise manner. **(A)** Effect of the overexpression of ScCTA1 and ScCTT1 catalases on the product titer. **(B)** Fermentation was conducted in YPD medium. WCA8, *INO2* gene overexpressed; WCA9, *HEM3* gene overexpressed; WCA10, *POS5* gene overexpressed; WCA11, *HAC1* gene overexpressed. The data are the averages of three separate experiments.

As shown in Figure 6A, we expressed *ScCTA1* from the *MET17* locus of WCA6 to construct the WCA7a strain. Overexpression of *ScCTA1* increased CA production to 7.57 mg/L and ferruginol production to 11.38 mg/L. Next, we expressed *ScCTT1* from the *MET17* locus of WCA6 to construct the WCA7b strain, which exhibited increases of CA and ferruginol production to 8.84 mg/L and 14.64 mg/L. However, when the two genes were simultaneously overexpressed, CA production was reduced. Therefore, the WCA7b strain was selected for subsequent experiments.

### Effect of Endoplasmic Reticulum Modification and Cofactor Metabolism Regulation on Carnosic Acid Synthesis

The subcellular organelles of eukaryotes have different functions, providing a unique physicochemical environment and critical functions for cell survival. For instance, the Endoplasmic Reticulum (ER) offers a microenvironment designed for rapid and precise protein processing (Malhotra et al., 2008). Accordingly, the ER volume is a crucial determinant of the protein folding capacity of cells (Schuck et al., 2009). The ER membrane is mainly constituted by phospholipids, and the transcription factor INO2 can activate the expression of related genes to promote phospholipid synthesis, thus increasing the area of the ER membrane. In a recent study, overexpression of *INO2* in yeast expanded the ER area, leading to a 71-fold increase in squalene production (Kim et al., 2019). As shown in Figure 6B, the *INO2* overexpression in strain WCA8 boosted the CA titer by 29%, reaching 11.42 mg/L, and the ferruginol titer was 20.25 mg/L.

Heme is the main cofactor of P450s, and modifying the endogenous heme synthesis pathway was reported as a viable approach to enhance the activity of P450s (Michener et al., 2012). We overexpressed the *HEM3* (heme synthase) gene in the WCA9 strain, which increased the CA titer by 50% to 17.12 mg/L. To increase the NADPH supply, we overexpressed the NADH kinase gene (*POS5*) (Miyagi et al., 2009) in WCA10, which increased the CA titer by 20%–20.54 mg/L, whereby the ferruginol titer also increased to 33.86 mg/L (Figure 6B). The transcription factor HAC1 can activate the transcription of proteins folding-related genes, activating the unfolded protein response (UPR) in the ER (Khan and Schroeder, 2008; Jonikas et al., 2009; Qu et al., 2020). To reduce the pressure caused by the expansion of the ER, we overexpressed the transcription factor HAC1 in WCA11, which increased the CA and ferruginol titers to 24.65 and 36.29 mg/L, corresponding to yields of 1.23 and 1.81 mg/g, respectively (Figure 6B).

### Carnosic Acid Production in a 5-L Bioreactor

**FIGURE 7:**
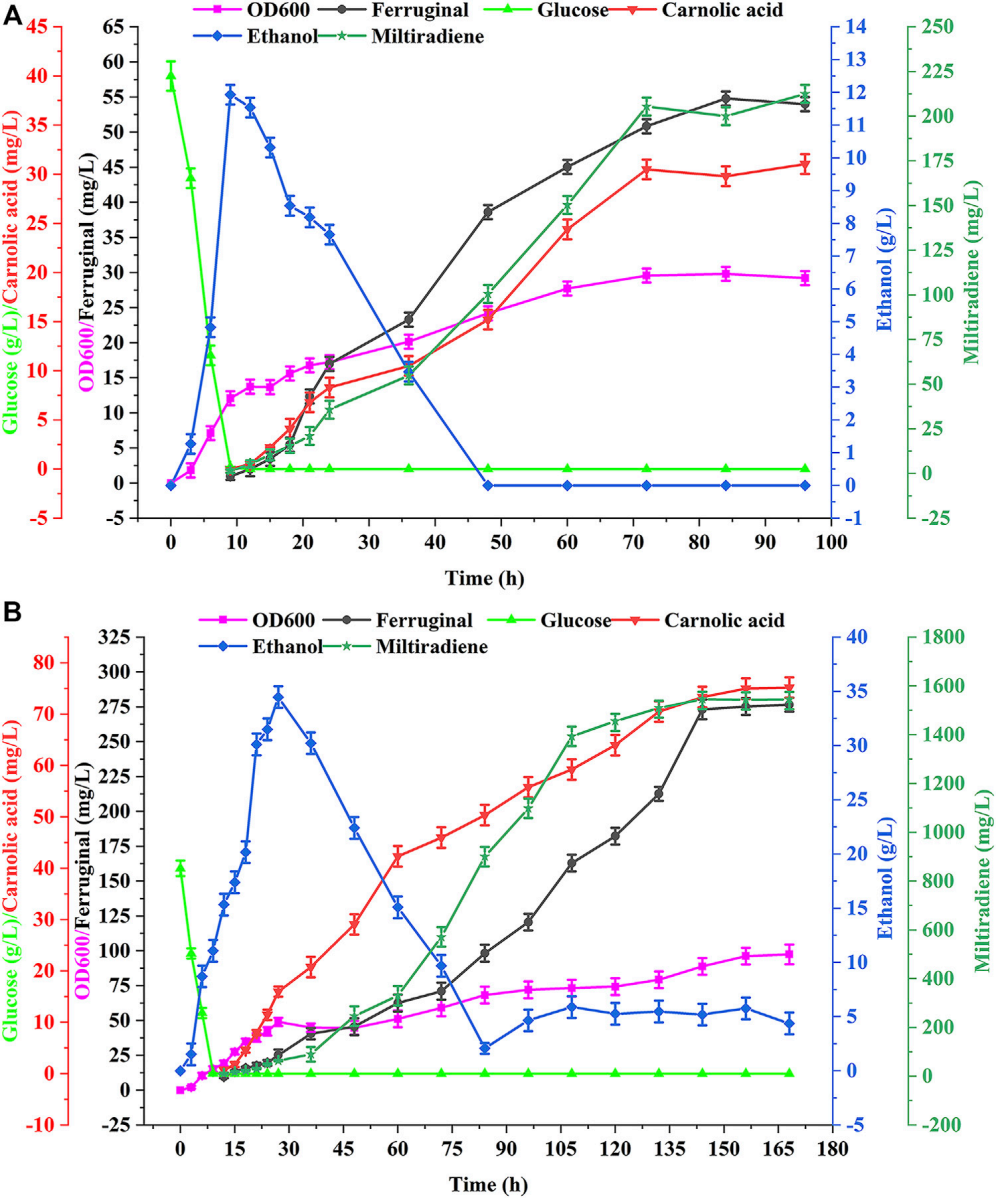
Production of CA in batch and fed-batch fermentations in a 5 L bioreactor. **(A)** Batch fermentation in a 5 L bioreactor using strain WCA11. The fermentation was conducted in YPD medium with 40 g/L glucose. **(B)** WCA11 fed-batch fermentation in a 5 L bioreactor. The data are the averages of three separate experiments.

We performed batch and fed-batch fermentation of the best strain WCA11 in a 5-L bioreactor to confirm the shake-flasks results. The residual glucose concentration, cell growth, ethanol concentration, and CA titer were evaluated during batch fermentation. Glucose was rapidly consumed within 12 h at the beginning of the fermentation (Figure 7A). At the same time, ethanol accumulation reached up to 11.93 g/L, after which ethanol was the carbon source and consumed after 48 h. CA production was detected every 12 h from the beginning of the fermentation, and reached a peak of 31.04 mg/L at 96 h. The titer of ferruginol and miltiradiene reached 54.80 and 212.45 mg/L, respectively. The final OD_600_ of the strain reached 29.23 (Figure 7A).

As shown in Figure 7B, the OD_600_ of WCA11 was 3.33-fold higher in fed-batch fermentation than in batch fermentation. The CA titer also increased considerably, reaching 75.18 mg/L, representing a 142.2% increase compared with batch fermentation, which is the highest CA titer reported in yeast to date, to our knowledge, (Table 1). The ferruginol and miltiradiene titers also increased to 276.58 and 1,543.27 mg/L, respectively. The two-stage feeding strategy therefore showed good results (Figure 7B).

**TABLE 1:** Carnosic acid titers in *S. cerevisiae* reported to date.

CA titer (mg/L)		References
Shake flask fermentation	5 L fed-batch fermentation	
1.00	—	Ignea et al. (2016)
2.74	—	Scheler et al. (2016)
18.09	—	Ignea et al. (2017)
24.65	75.18	This study

## Discussion

Diterpenoids have diverse structures and notable biological activities. However, the yield of traditional plant extraction is generally poor since the relevant compounds are present in low quantities as secondary metabolites. The phenolic tricyclic diterpene CA has valuable pharmacological and biological activities. Recently, many studies have been devoted to analyzing the synthesis pathway of CA. However, there are few studies on combinatorial engineering strategies to produce CA, and its high-level production in microorganisms is still challenging.

The spontaneous oxidation of miltiradiene into abietatriene is the subject of controversy. The *CYP76AH24* enzyme reportedly catalyzes the oxidation of the labdane skeleton at C-12 and C-11 to produce 11-hydroxy-ferruginol (Ignea et al., 2016). *CYP76AH1* can directly convert miltiradiene into abietatriene (Guo et al., 2013). In our study, the co-expression of *CYP76AH24* and *CYP76AH1* boosted the output of CA by more than 15 times compared with *CYP76AH24* alone, indicating that spontaneous oxidation is a rate-limiting step to some extent. It is also possible that *CYP76AH24* alone is less efficient than in the presence of *CYP76AH1*. Indeed, *CYP76AH24* would have to introduce two hydroxyl groups, whereas *CYP76AH1* introduces only one hydroxyl group, and its product can then serve as a substrate for *CYP76AH24*. However, because we could not source an authentic reference standard of 11-hydroxy-ferruginol, its production could not be confirmed. Although we attempted to identify 11-hydroxy-ferruginol among the new LC-MS peaks and found that the mass spectrum of a peak was similar to a published mass spectrum of 11-hydroxy-ferruginol, because there was no standard, a definitive confirmation could not be obtained (Supplementary Figure S7). We also detected several peaks, which may have been degradation products of 11-hydroxy-ferruginol or other by-products.

Overexpression of cytochrome B5 (Cytb5) increased the production of artemisinin (Paddon et al., 2013). In our study, the expression of Cytb5 from *S. pomifera* caused a greater than 3-fold increase in CA production, proving that Cytb5 could effectively improve the catalytic efficiency of P450 enzymes. Multicopy integration is a common strategy in microbial biosynthesis of natural products (Zhang et al., 2018). After applying the multicopy integration strategy, CA production was nearly doubled in this study. Reasonable regulation of the supply of cofactors can increase metabolic fluxes and promote product accumulation (Wang et al., 2017). The cofactor regulation strategy used in this study increased CA production more than 2-fold.

The combined engineering strategy used in this study gradually increased the production of CA (Table 2). During this process, the output of the intermediate product miltiradiene gradually decreased (Supplementary Table S5), but the remaining titer was still 109.89 mg/L. Therefore, engineering CYP76AH1, or mining other enzymes with higher activities may be an effective way to increase the production of CA. Recently, researchers used protein engineering to functionally optimize CYP76AH1, and they designed a mutant that catalyzes the highly efficient production of 11-hydroxy-ferruginol in yeast (Mao et al., 2020). Based on the findings of this study, this strategy may lead to a substantial increase of CA production. Additionally, with the gradual increase of CA production, the production of intermediate ferruginol also gradually increased (Supplementary Table S5). The titer of ferruginol increased to 276.58 mg/L in fed-batch fermentation. In comparison, the yield of CA was 75.18 mg/L, which suggests that the low conversion rate of the intermediate ferruginol is a critical problem that limits the final CA yield. Exploring a more optimal fed-batch fermentation strategy may improve the yield of the product CA. Furthermore, Engineering CYP76AH24 and CYP76AK6 to improve their catalytic activities may be a practical solution. Finally, based on the findings of this study related to fusion protein design and signal peptide truncation, the selection of linkers and truncations can still be further studied to obtain a higher CA titer.

**TABLE 2:** Overview of carnosic acid titer improvement.

Strain	Modified genes	CA titer (mg/L)	Fold improvement (from previous step)	Fold improvement (total)
WCA1a	*AtCPR, CYP76AH24, CYP76AK6*	0.03 ± 0.002	—	—
WCA1b	*GuCPR, CYP76AH24, CYP76AK6*	0.04 ± 0.003	—	—
WCA1c	*SmCPR, CYP76AH24, CYP76AK6*	0.05 ± 0.003	—	—
WCA2	***SmCPR*** (2 copies)*, CYP76AH24, CYP76AK6,* ***CYP76AH1***	0.82 ± 0.04	16.20	16.20
WCA3	*SmCPR* (2 copies)*,CYP76AH24, CYP76AK6, CYP76AH1,* ***t28SpCyb5***	2.62 ± 0.37	3.23	52.4
WCA4c	*SmCPR, CYP76AH24, CYP76AK6, CYP76AH1,* ***SmCPR-GGG-t28SpCyb5***	3.17 ± 0.29	3.91	63.40
WCA5	*SmCPR, CYP76AH24, CYP76AK6, CYP76AH1* (**multiple copies**)*, SmCPR-GGG-t28SpCyb5* (**multiple copies**)	4.30 ± 0.72	1.36	86.00
WCA6	*SmCPR,CYP76AH24* (**multiple copies**)*, CYP76AK6* (**multiple copies**)*, CYP76AH1* (multiple copies)*, SmCPR-GGG-t28SpCyb5 (*multiple copies)	6.20 ± 1.38	1.44	124.00
WCA7b	*SmCPR, CYP76AH24* (multiple copies)*, CYP76AK6* (multiple copies)*, CYP76AH1* (multiple copies)*, SmCPR-GGG-t28SpCyb5* (multiple copies)*,* ***ScCTT1***	8.84 ± 1.67	1.43	176.80
WCA8	*SmCPR, CYP76AH24* (multiple copies)*, CYP76AK6* (multiple copies)*, CYP76AH1* (multiple copies)*, SmCPR-GGG-t28SpCyb5* (multiple copies)*, ScCTT1,* ***INO2***	11.42 ± 1.54	1.29	228.40
WCA9	*SmCPR, CYP76AH24* (multiple copies)*, CYP76AK6* (multiple copies)*, CYP76AH1* (multiple copies)*, SmCPR-GGG-t28SpCyb5* (multiple copies)*, ScCTT1, INO2,* ***HEM3***	17.12 ± 1.74	1.50	342.40
WCA10	*SmCPR, CYP76AH24* (multiple copies)*, CYP76AK6* (multiple copies)*, CYP76AH1 (multiple copies), SmCPR-GGG-t28SpCyb5* (multiple copies)*, ScCTT1, INO2, HEM3,* ***POS5***	20.54 ± 1.61	1.20	410.80
WCA11	*SmCPR, CYP76AH24* (multiple copies)*, CYP76AK6* (multiple copies)*, CYP76AH1* (multiple copies)*, SmCPR-GGG-t28SpCyb5* (multiple copies)*, ScCTT1, INO2, HEM3, POS5,* ***HAC1***	24.65 ± 1.42	1.20	493.00

Note: All the strains listed in the table additionally expressed the BTS1-GGGS-ERG20(F96C) and tCPS-GSTSSGSSG-tKSL, fusion proteins. The gene modified after the previous step is indicated in bold.

In summary, we described a combined engineering strategy to gradually increase the CA output. As a result, the CA titer reached 24.65 mg/L in shake flasks and 75.18 mg/L in 5 L fed-batch fermentation. This engineering strategy has reference value for improving the production of other diterpenoids in microbial cell factories.

## Data Availability

The original contributions presented in the study are included in the article/Supplementary Material, further inquiries can be directed to the corresponding author.
