# Supplementary material for: Metabolic Engineering of Saccharomyces cerevisiae for Heterologous Carnosic Acid Production
Source: Front Bioeng Biotechnol. 2022 Jun 2;10:916605. doi: 10.3389/fbioe.2022.916605 (PMC9201568; doi:10.3389/fbioe.2022.916605)
Supplement: Supplementary file 1 [file DataSheet1.docx]

**Table S1.** Strains and plasmids used in this work.

| Strains | Chassis strains | Characteristics | Source |
| --- | --- | --- | --- |
| 3HP-F | W303-1a | ade2::P_PGK1_-tHMG1-T_PGK1_*，*P_TDH3_-ERG20-T_ERG20_; P_ERG9_::P_HXT1_-ERG9-PEST | (Zhang et al., 2020) |
| WM1 | 3HP-F | ura3::*P_PGK1_-CPS-T_ADH2_，P_TDH3_-KSL-T_TDH2_* HIS3 | This work |
| WM2 | 3HP-F | ura3::*P_PGK1_-tCPS-T_ADH2_，P_TDH3_-tKSL-T_TDH2_* HIS3 | This work |
| WM2a | 3HP-F | ura3::*P_PGK1_-CPS-**mcherry-T_ADH2_* HIS3 | This work |
| WM2b | 3HP-F | ura3::*P_PGK1_-tCPS-mcherry-T_ADH2_* HIS3 | This work |
| WM2c | 3HP-F | ura3::*P_PGK1_-KSL-mcherry-T_ADH2_* HIS3 | This work |
| WM2d | 3HP-F | ura3::*P_PGK1_-tKSL-mcherry-T_ADH2_* HIS3 | This work |
| WM3a | 3HP-F | ura3::*P_PGK1_-tCPS-linker1-tKSL-T_TDH2_* HIS3 | This work |
| WM3b | 3HP-F | ura3::*P_PGK1_-tCPS-linker2-tKSL-T_TDH2_* HIS3 | This work |
| WM3c | 3HP-F | ura3::*P_PGK1_-tCPS-linker3-tKSL-T_TDH2_* HIS3 | This work |
| WM3d | 3HP-F | ura3::*P_PGK1_-tCPS-linker4-tKSL-T_TDH2_* HIS3 | This work |
| WM3e | 3HP-F | ura3::*P_PGK1_-tCPS-tKSL-T_TDH2_* HIS3 | This work |
| WM3f | 3HP-F | ura3::*P_PGK1_- tKSL-linker1-tCPS -T_TDH2_*  HIS3 | This work |
| WM4 | 3HP-F | Ho:: *P_TDH3_-BTS1-GGGS-ERG20(F96C)-T_ERG20,_ P_PGK1_-tCPS-linker1-tKSL-T_TDH2_*  TRP1 | This work |
| WCA1a | WM4 | trp1:: *P_PGK1_-ATR1-T_CYC1_, P_TEF1_-CYP76AH24-T_TDH2_, P_TDH3_-CYP76AK6-T_ADH2_*  URA3 | This work |
| WCA1b | WM4 | trp1:: *P_PGK1_-GuCPR-T_CYC1_, P_TEF1_-CYP76AH24-T_TDH2_, P_TDH3_-CYP76AK6-T_ADH2_*  URA3 | This work |
| WCA1c | WM4 | trp1:: *P_PGK1_-SmCPR-T_CYC1_, P_TEF1_-CYP76AH24-T_TDH2_, P_TDH3_-CYP76AK6-T_ADH2_*  URA3 | This work |
| WCA2 | WCA1c | ade2:: *P_TEF1_- SmCPR-T_CYC1_, P_TDH3_-CYP76AH1-T_ADH2_*  LEU2 | This work |
| WCA3 | WCA2 | ura3:: *P_PGK1_-SpCyb5-T_ADH2_*  HIS3 | This work |
| WCA4a | WCA1c | ade2:: *P_TEF1_- CYP76AH1-* *GGG-t60SmCPR-T_CYC1_, P_PGK1_-* *SpCyb5-T_ADH2_*  LEU2 | This work |
| WCA4b | WCA1c | ade2:: *P_TEF1_- SmCPR- T_CYC1_,GGG P_TDH3_-CYP76AH1-GGG-t28SpCyb5-T_ADH2_*  LEU2 | This work |
| WCA4c | WCA1c | ade2:: *P_TEF1_- SmCPR-* *GGG- t28SpCyb5-T_CYC1_, P_TDH3_-* *CYP76AH1-T_ADH2_*  LEU2 | This work |
| WCA4d | WCA1c | ade2:: *P_TEF1_- CYP76AH1-GGG-t60SmCPR-* *GGG- t28SpCyb5-T_CYC1_*  LEU2 | This work |
| WCA5 | WCA4c | *δ2:: P_PGK1_- SmCPR-* *GGG- t28SpCyb5-T_CYC1_, P_TDH3_-* *CYP76AH1-T_ADH2_*  G418 | This work |
| WCA6 | WCA5 | rDNA:: *P_PGK1_- CYP76AH24-T_TDH2_, P_TDH3_-* *CYP76AK6-T_CYC1_*  HIS3 | This work |
| WCA7a | WCA6 | met17:: *P_PGK1_- ScCTA1-T_TDH2_*  HYGR | This work |
| WCA7b | WCA6 | met17:: *P_PGK1_- ScCTT1-T_TDH2_*  HYGR | This work |
| WCA7c | WCA6 | met17::*P_PGK1_-ScCTA1-T_TDH2_,P_TDH3_-ScCTT1-T_ADH2_*  HYGR | This work |
| WCA8 | WCA7b | ura3::*P_PGK1_-INO2-T_TDH2_*  MET17 | This work |
| WCA9 | WCA7b | ura3:: *P_PGK1_-INO2-T_TDH2_, P_TDH3_-HEM3-T_CYC1_* MET17 | This work |
| WCA10 | WCA7b | ura3:: *P_PGK1_-INO2-T_TDH2_, P_TDH3_-HEM3-T_CYC1_, P_TEF1_-POS5-T_TDH2_*  MET17 | This work |
| WCA11 | WCA7b | ura3:: *P_PGK1_-INO2-T_TDH2_, P_TDH3_-HEM3-T_CYC1_, P_TEF1_-POS5-T_TDH2_,P_PGK1_-HAC1-T_TDH2_* MET17 | This work |

**Table S2**. All the primers used in this study.

| Name | Sequences 5’-3’ |
| --- | --- |
| URA3U-F | GACGTTGAAATTGAGGCTACTGCG |
| PGK1p-URA3U-R | ATAATATCTGTGCGTGACCTAATGCTTCAACTAAC |
| URA3U-PGK1p-F | GTTGAAGCATTAGGTCACGCACAGATATTATAACATC |
| SmCPS-PGK1p-R | CAAAGAGGCCATCATTGTTTTATATTTGTTGTAAAAAGTAG |
| PGK1p-SmCPS-F | ATATAAAACAATGATGGCCTCTTTGTCCTCTAC |
| ADH2t-SmCPS-R | ACATAAGAGATCCGCTTAGGCAACTGGCTCAAACAAAA |
| SmCPS-ADH2t-F | TGGAGCCAGTTGCCTAAGCGGATCTCTTATGTCTTTACGATT |
| ADH2t-R-TDH3p | ATTCAACGCTAGTATTAGAATTATATAACTTGATGAGATGAGATGAG |
| ADH2t-TDH3p-F | AGTTATATAATTCTAATACTAGCGTTGAATGTTAGCGTCA |
| TDH3p-R-SmKSL | AAAAGCCAAGGACATCCATTTTGTTTGTTTATGTGTG |
| TDH3p-SmKSL-F | AAACAAACAAAATGGATGTCCTTGGCTTTTAATCCAG |
| SmKSL-R-TDH2t | GTAACTTAAGGAGTTATTACTTACCTCTGACATTGTTGGC |
| SmKSL-TDH2t-F | GTCAGAGGTAAGTAATAACTCCTTAAGTTACTTTAATGAT |
| TDH2t-R-HIS3 | CTGCAGGCATGCAAGCGCGAAAAGCCAATTAGTGTGATACT |
| TDH2t-HIS3-F | TAATTGGCTTTTCGCGCTTGCATGCCTGCAGGTC |
| HIS3-R-URA3D | TGTAGAGACCACATCAGCACGTGATGAATTCGAGCTCG |
| HIS3-URA3D-F | AATTCATCACGTGCTGATGTGGTCTCTACAGGATCTGACA |
| URA3D-R | CAAGCCTTGTCCCAAGGCAGCG |
| check-SmCPS-F | GACTGATAAGGGTGTGTTCTCTGG |
| check-ADH2t-R | GTTTAGAGGAATGGGTACAACTCACAG |
| check-SmKSL-F | TCTGAAGATTTCTTGGCTTTCGCT |
| check-TDH2t-R | AAAACTTCCAAAAAAATCGAATCCCTGA |
| PGK1p-R-tSmCPS | AGGAACCCATCCAAGCCATTGTTTTATATTTGTTGTAAAAAGTAGATAA |
| PGK1p-tSmCPS-F | CAAATATAAAACAATGGCTTGGATGGGTTCCTCTTCTAAAA |
| tSmCPS-R-ADH2t | ACATAAGAGATCCGCTTAGGCAACTGGCTCAAACAAAACC |
| tSmCPS-ADH2t-F | TGAGCCAGTTGCCTAAGCGGATCTCTTATGTCTTTACGATT |
| TDH3p-R-tSmKSL | AGTAGTCAAGTTACACATTTTGTTTGTTTATGTGTGTTTA |
| TDH3p-tSmKSL-F | TAAACAAACAAAATGTGTAACTTGACTACTACTGACTTGA |
| tSmKSL-R-TDH2t | GTAACTTAAGGAGTTATTACTTACCTCTGACATTGTTGGCA |
| tSmKSL-TDH2t-F | GTCAGAGGTAAGTAATAACTCCTTAAGTTACTTTAATGAT |
| PGK1p-KSL-F | GGAAGTAATTATCTACTTTTTACAACAAATAATGTCCTTGGCTTTTAATCCAGC |
| PGK1p-R-KSL | GCTGGATTAAAAGCCAAGGACATTATTTGTTGTAAAAAGTAGATAATTACTTCC |
| PGK1p-tKSL-F | GGAAGTAATTATCTACTTTTTACAACAAATAATGTGTAACTTGACTACTACTGACTTGATG |
| PGK1p-R-tKSL | CATCAAGTCAGTAGTAGTCAAGTTACACATTATTTGTTGTAAAAAGTAGATAATTACTTCC |
| KSL-MCH-F | GTTGCCAACAATGTCAGAGGTAAGATGGTGAGCAAGGGCGAG |
| KSL-R-MCH | CTCGCCCTTGCTCACCATCTTACCTCTGACATTGTTGGCAAC |
| CPS-MCH-F | GGTTTTGTTTGAGCCAGTTGCCATGGTGAGCAAGGGCGAG |
| CPS-R-MCH | CTCGCCCTTGCTCACCATGGCAACTGGCTCAAACAAAACC |
| MCH-ADH2t-F | CATGGACGAGCTGTACAAGTAGGCGGATCTCTTATGTCTTTACGATTTAT |
| MCH-R-ADH2t | ATAAATCGTAAAGACATAAGAGATCCGCCTACTTGTACAGCTCGTCCATG |
| ADH2t-R-HIS | GACCTGCAGGCATGCAAGCTAGAATTATATAACTTGATGAGATGAGATGAGTAAATG |
| ADH2t-HIS-F | CATTTACTCATCTCATCTCATCAAGTTATATAATTCTAGCTTGCATGCCTGCAGGTC |
| tSmCPS-linker1-R | ACAACCAGAAGAACCAGAAGAAGTAGAACCGGCAACTGGCTCAAACAAAACCTTA |
| linker1-tSmKSL-F | GCCGGTTCTACTTCTTCTGGTTCTTCTGGTTGTAACTTGACTACTACTGACTTGA |
| tSmCPS-linker2-R | AGTTACAACCAGAAGAAGTAGAACCGGCAACTGGCTCAAACAAAACCTTA |
| linker2-tSmKSL-F | GTTGCCGGTTCTACTTCTTCTGGTTGTAACTTGACTACTACTGACTTGA |
| tSmCPS-linker3-R | GTCAAGTTACAACCACCACCGGCAACTGGCTCAAACAAAACCTTA |
| linker3-tSmKSL-F | AGCCAGTTGCCGGTGGTGGTTGTAACTTGACTACTACTGACTTGA |
| tSmCPS-linker4-R | CAAGTCAGTAGTAGTCAAGTTACAAGAACCACCACCGGCAACTGGCTCAAACAAAACCTTAA |
| linker4-tSmKSL-F | AGGTTTTGTTTGAGCCAGTTGCCGGTGGTGGTTCTTGTAACTTGACTACTACTGACTTGA |
| tSmCPS-nolinker-R | TCAGTAGTAGTCAAGTTACAGGCAACTGGCTCAAACAAAACCTTA |
| nolinker-tSmKSL-F | AGGTTTTGTTTGAGCCAGTTGCCTGTAACTTGACTACTACTGACTTGA |
| HOU-F | TCCCAGGCGTAGAACAGTTTATCAG |
| HOU-R-TDH3p | TCAACGCTAGTATGATCCAAGCTATCTACTGAG |
| HOU-TDH3p-F | AGATAGCTTGGATCATACTAGCGTTGAATGTTAG |
| BTS1-TDH3-R | ATCTTGGCCTCCATTTTGTTTGTTTATGTGTGTTTATTC |
| TDH3-BTS1-F | CATAAACAAACAAAATGGAGGCCAAGATAGATGAG |
| lingker-BTS1-R | TGAAGCCATAGAACCACCACCCAATTCGGATAAGTGGTCTAT |
| lingker-ERG20-F | GGTGGTGGTTCTATGGCTTCAGAAAAAGAAATT |
| F96C-R | TCGGCGACCAAGCAGTAAGCCTGCAACAACTC |
| F96C-F | CAGGCTTACTGCTTGGTCGCCGAT |
| ERG20t-R-PGK1p | GTTATAATATCTGTGCGTCAATGTTCGAGAGATGAGGTCGTTG |
| ERG20t-PGK1p-F | CTCATCTCTCGAACATTGACGCACAGATATTATAACATCTGCATAATAGG |
| TDH2t-R-TRP1 | AGGCATGCAAGCTTGGCGAAAAGCCAATTAGTGTGATACTAAG |
| TDH2t-TRP1-F | TAATTGGCTTTTCGCCAAGCTTGCATGCCTGCAGG |
| HOD-TRP1-R | TGACATACCAAGAACAAAGGCAGCTTGGAGTATGTCT |
| TRP1-HOD-F | TCCAAGCTGCCTTTGTTCTTGGTATGTCAGCTACTGTG |
| HOD-R | CACTTCACGTGCTTCTGGTACATACTTG |
| TRP1U-F | GGAAGAGGAGTAGGGAATATTACTGGC |
| TRP1U-R-URA3 | ACTTCGTATAATGTATGCCTGCAAGCCGCAAACTTTCAC |
| TRP1U-URA3-F | GAAAGTTTGCGGCTTGCAGGCATACATTATACGAAGTTATCAGGGTCC |
| URA3-R-PGK1p | GTTATAATATCTGTGCGTATAATGTATGCTATACGAAGTTATGGGTAATAACTG |
| URA3-PGK1p-F | TCGTATAGCATACATTATACGCACAGATATTATAACATCTGCATAATAGG |
| PGK1p-R-ATR1 | GTATAATGCAGAAGTCATCATTGTTTTATATTTGTTGTAAAAAGTAGATAATTACTTCCTTG |
| PGK1p-ATR1-F | AACAAATATAAAACAATGATGACTTCTGCATTATACGCATCAGAC |
| ATR1-R-CYC1t | ACATAACTAATTACATGATTACCAGACGTCCCTCAAGTACC |
| ATR1-CYC1t-F | TTGAGGGACGTCTGGTAATCATGTAATTAGTTATGTCACGCTTACATTCA |
| CYC1t-R-TEF1p | GTGTGTGGGGGATCACTTGCAAATTAAAGCCTTCGAGCGTC |
| CYC1t-TEF1p-F | TCGAAGGCTTTAATTTGCAAGTGATCCCCCACACACCATA |
| TEF1p-R-76AH24 | AGAACCCAATTGCAACATCCATTTTGTAATTAAAACTTAGATTAGATTGCTATGCT |
| TEF1p-76AH24-F | GTTTTAATTACAAAATGGATGTTGCAATTGGGTTCTCAACCA |
| 76AH24-R-TDH2t | AGTAACTTAAGGAGTTAAATTTAAGCCTTGATTGGAACGATTTTCAATG |
| 76AH24-TDH2t-F | GTTCCAATCAAGGCTTAAATTTAACTCCTTAAGTTACTTTAATGATTTAGTTTTTATTATTAATAATTCATG |
| TDH2t-R-TDH3p | AACATTCAACGCTAGTATGCGAAAAGCCAATTAGTGTGATACTAAG |
| TDH2t-TDH3p-F | CACTAATTGGCTTTTCGCATACTAGCGTTGAATGTTAGCGTCAA |
| TDH3p-R-76AK6 | CAAGATCAAGACCTGCATTTTGTTTGTTTATGTGTGTTTATTCGAAACTAAGT |
| TDH3p-76AK6-F | CACACATAAACAAACAAAATGCAGGTCTTGATCTTGTTGTCTTT |
| 76AK6-R-ADH2t | AAGACATAAGAGATCCGCTCAGACCTTGATTGGAATGGCTC |
| 76AK6-ADH2t-F | ATTCCAATCAAGGTCTGAGCGGATCTCTTATGTCTTTACGATTTATAGTTT |
| ADH2t-R-TRP1D | ATAACCTATTTCTTAGCATAGAATTATATAACTTGATGAGATGAGATGAGTAAATGAC |
| ADH2t-TRP1D-F | TCAAGTTATATAATTCTATGCTAAGAAATAGGTTATTACTGAGTAGTATTTATTTAAGTATT |
| TRP1D-R | CATTGATGAGGCAACGCTAATTATCAAC |
| PGK1p-R-GuCPR | AGAGTTGGAGGTCATCATTGTTTTATATTTGTTGTAAAAAGTAGATAATTACTTCCTTG |
| PGK1p-GuCPR-F | AAATATAAAACAATGATGACCTCCAACTCTGATTTGGTTAG |
| GuCPR-R-CYC1t | TAACTAATTACATGATCACCAAACATCCCTCAAGTATCTAC |
| GuCPR-CYC1t-F | AGGGATGTTTGGTGATCATGTAATTAGTTATGTCACGCTTACATTCA |
| PGK1p-R-SmCPR | AGAAGATGGTTCCATCATTGTTTTATATTTGTTGTAAAAAGTAGATAATTACTTCCTTG |
| PGK1p-SmCPR-F | AAATATAAAACAATGATGGAACCATCTTCTAAAAAATTGTCTCC |
| SmCPR-R-CYC1t | TAACTAATTACATGACCAAACATCTCTCAAATATCTACCAGTAGTTT |
| SmCPR-CYC1t-F | TTGAGAGATGTTTGGTCATGTAATTAGTTATGTCACGCTTACATTCA |
| ADE2U-F | TAACGCCGTATCGTGATTAACGTATTAC |
| ADE2U-R-TEF1p | TGTGGGGGATCACTTATACATGAAATGCTCCATAATATTGTCCATTTAG |
| ADE2U-TEF1p-F | GAGCATTTCATGTATAAGTGATCCCCCACACACC |
| TEF1p-R-SmCPR | AGAAGATGGTTCCATCCATTTTGTAATTAAAACTTAGATTAGATTGCTATGCT |
| TEF1p-SmCPR-F | TTAATTACAAAATGGATGGAACCATCTTCTAAAAAATTGTCTCC |
| TDH3p-R-76AH1 | TGGAAAAGAATCCATTTTGTTTGTTTATGTGTGTTTATTCGAAACTAAG |
| TDH3p-76AH1-F | ACATAAACAAACAAAATGGATTCTTTTCCATTGTTGGCTG |
| 76AH1-R-ADH2t | ACATAAGAGATCCGCTTAAGATTTAACAATTGGAATAATTCTCAATGGAACA |
| 76AH1-ADH2t-F | ATTGTTAAATCTTAAGCGGATCTCTTATGTCTTTACGATTTATAGTT |
| ADH2t-R-LEU2 | TGCAGGCATGCAAGCTAGAATTATATAACTTGATGAGATGAGATGAGTAAATGAC |
| ADH2t-LEU2-F | AGTTATATAATTCTAGCTTGCATGCCTGCAGGT |
| LEU2-R-ADE2D | CATCTAGACAAGAACACCCGGGATAACTTCGTATAATGTATG |
| LEU2-ADE2D-F | GAAGTTATCCCGGGTGTTCTTGTCTAGATGGAGTAGATTCTTTACATTC |
| ADE2D-R | CGCTATCCTCGGTTCTGCATT |
| PGK1p-R-SpCytb5 | ATGAGATTTTGCCATCATTGTTTTATATTTGTTGTAAAAAGTAGATAATTACTTCCTTG |
| PGK1p-SpCytb5-F | ACAACAAATATAAAACAATGATGGCAAAATCTCATACTTTTGAAGAAGTTG |
| SpCytb5-R-ADH2t | ACATAAGAGATCCGCTTATTTTTCTTTAGTATACAATCTAACAGCAAAAGCC |
| SpCytb5-ADH2t-F | ACTAAAGAAAAATAAGCGGATCTCTTATGTCTTTACGATTTATAGTT |
| TEF1p-R-76AH1 | TGGAAAAGAATCCATCCATTTTGTAATTAAAACTTAGATTAGATTGCTATGCT |
| TEF1p-76AH1-F | TTAATTACAAAATGGATGGATTCTTTTCCATTGTTGGCTG |
| AH1-R-linker3-t60SmCPR | AGCAGTTCTTCTCATACCACCACCAGATTTAACAATTGGAATAATTCTCAATGGAACAG |
| AH1-linker3-t60SmCPR-F | CCAATTGTTAAATCTGGTGGTGGTATGAGAAGAACTGCTGGTTCTGC |
| CYC1t-R-PGK1p | GTTATAATATCTGTGCGTGCAAATTAAAGCCTTCGAGCGTCCC |
| CYC1t-PGK1p-F | TCGAAGGCTTTAATTTGCACGCACAGATATTATAACATCTGCATAAT |
|  |  |
| AH1-R-linker3- t28SpCytb5 | ATGAGATTTTGCCATACCACCACCAGATTTAACAATTGGAATAATTCTCAATGGAACAG |
| AH1-linker3- t28SpCytb5-F | CCAATTGTTAAATCTGGTGGTGGTATGGCAAAATCTCATACTTTTGAAGAAGTTG |
| t28SpCytb5-R-ADH2t | ACATAAGAGATCCGCTTATTCAGGAGTTTTATCAGGATTATAAGATGGTT |
| t28SpCytb5-ADH2t-F | AAAACTCCTGAATAAGCGGATCTCTTATGTCTTTACGATTTATAGTT |
| SmCPR-R-linker3- t28SpCytb5 | ATGAGATTTTGCCATACCACCACCAACATCTCTCAAATATCTACCAGTAGTTTGC |
| SmCPR-linker3- t28SpCytb5-F | TATTTGAGAGATGTTGGTGGTGGTATGGCAAAATCTCATACTTTTGAAGAAGTTG |
| t28SpCytb5-R-CYC1t | TAACTAATTACATGATTATTCAGGAGTTTTATCAGGATTATAAGATGGTT |
| t28SpCytb5-CYC1t-F | AAAACTCCTGAATAATCATGTAATTAGTTATGTCACGCTTACATTCA |
| TEF1p-R-t60SmCPR | AGCAGTTCTTCTCATCCATTTTGTAATTAAAACTTAGATTAGATTGCTATGCT |
| TEF1p-t60SmCPR-F | TTAATTACAAAATGGATGAGAAGAACTGCTGGTTCTGC |
| ADH2t-R-TEF1p | TGTGGGGGATCACTTTAGAATTATATAACTTGATGAGATGAGATGAGTAAATGAC |
| ADH2t-TEF1p-F | AGTTATATAATTCTAAAGTGATCCCCCACACACCA |
| SpCytb5-R-CYC1t | TAACTAATTACATGATTATTTTTCTTTAGTATACAATCTAACAGCAAAAGCC |
| SpCytb5-CYC1t-F | ACTAAAGAAAAATAATCATGTAATTAGTTATGTCACGCTTACATTCA |
| δ2-F | GCTTCGGTTACTTCTAAGGAAGTCC |
| δ2-R-PGK1p | ATAATATCTGTGCGTTTGGAAAGTCATTAGGTGAGGTTAACATT |
| δ2-PGK1p-F | CTAATGACTTTCCAAACGCACAGATATTATAACATCTGCATAATAG |
| ADH2t-R-G418 | TACGAAGCTTCAGCTTAGAATTATATAACTTGATGAGATGAGATGAGTAAATGAC |
| ADH2t-G418-F | AGTTATATAATTCTAAGCTGAAGCTTCGTACGCTG |
| G418-R-δ1 | ATTTCTATTCCAACAAGTATAGCGACCAGCATTCACATAC |
| G418-δ1-F | GCTGGTCGCTATACTTGTTGGAATAGAAATCAACTATCATCTACTAACTAG |
| rDNA2-F-1 | CCGGGGCACCTGTCACT |
| rDNA2-R-PGK1p | ATAATATCTGTGCGTTTTCCTCTAATCAGGTTCCACCAAAC |
| rDNA2-PGK1p-F | CCTGATTAGAGGAAAACGCACAGATATTATAACATCTGCATAATAGG |
| PGK1p-R-76AH24 | ACCCAATTGCAACATCATTGTTTTATATTTGTTGTAAAAAGTAGATAATTACTTCCTT |
| PGK1p-76AH24-F | AAATATAAAACAATGATGTTGCAATTGGGTTCTCAACC |
| 76AK6-R-CYC1t | TAACTAATTACATGATCAGACCTTGATTGGAATGGCTC |
| 76AK6-CYC1t-F | CCAATCAAGGTCTGATCATGTAATTAGTTATGTCACGCTTACATTCA |
| CYC1t-R-HiS3 | TGCAGGCATGCAAGCGCAAATTAAAGCCTTCGAGCGTC |
| CYC1t-HiS3-F | AAGGCTTTAATTTGCGCTTGCATGCCTGCAGGT |
| HIS3-R-rDNA1 | TACGTTTGCTACTCTCATAGCACGTGATGAATTCGAGCTC |
| HIS3-rDNA1-F | TCGAATTCATCACGTGCTATGAGAGTAGCAAACGTAAGTCTAAAGGT |
| rDNA1-R-1 | GCGGAAAATACGGAAACGCG |
| METU-F | GGCACCTTGTCCAATTGAACAC |
| METU-R-HYGR | CTGGGCCTCCATGTCTCTGGATTGTCACCTTCAACAAATCTAG |
| METU-HYGR-F | AGGTGACAATCCAGAGACATGGAGGCCCAGAATACC |
| HYGR-R-PGK1p | ATAATATCTGTGCGTACAGCAGTATAGCGACCAGCA |
| HYGR-PGK1p-F | TCGCTATACTGCTGTACGCACAGATATTATAACATCTGCATAATAGG |
| PGK1p-R-ScCTA1 | AATCTAGTACTATTTCATTGTTTTATATTTGTTGTAAAAAGTAGATAATTACTTCCTTG |
| PGK1p-ScCTA1-F | AAATATAAAACAATGAAATAGTACTAGATTCATATGATTGTTATAATAATGGTGAATTG |
| ScCTA1-R-TDH2t | CTTAAGGAGTTAAATCACTCAGCTTATTATAGACTTCTGGTTTTGAT |
| ScCTA1-TDH2t-F | ATAATAAGCTGAGTGATTTAACTCCTTAAGTTACTTTAATGATTTAGTTTTTATTATTAATAATTCATG |
| TDH2t-R-METD | ACTCATTACGCACACGCGAAAAGCCAATTAGTGTGATACTAAGT |
| TDH2t-METD-F | TAATTGGCTTTTCGCGTGTGCGTAATGAGTTGTAAAATTATGTATAAACC |
| METD-R | ACGAGGAGAACTAGTATGTCCTGG |
| HYGR-R-TDH3p | ATTCAACGCTAGTATACAGCAGTATAGCGACCAGCA |
| HYGR-TDH3p-F | TCGCTATACTGCTGTATACTAGCGTTGAATGTTAGCGTCAAC |
| TDH3p-R-ScCTT1 | TCTTTTATGTTTTGATTTGTTTGTTTATGTGTGTTTATTCGAAACTAAGT |
| TDH3p-ScCTT1-F | ACATAAACAAACAAATCAAAACATAAAAGAAAAGAAAAAAATTAAATTGAATAAGTCAG |
| ScCTT1-R-ADH2t | ACATAAGAGATCCGCACTTTGGTAAGATATTATTATAACAGAAAGAATTTAAAGTTTTCT |
| ScCTT1-ADH2t-F | ATATCTTACCAAAGTGCGGATCTCTTATGTCTTTACGATTTATAGTT |
| ADH2t-R-METD | ACTCATTACGCACACTAGAATTATATAACTTGATGAGATGAGATGAGTAAATGAC |
| ADH2t-METD-F | TCTCATCAAGTTATATAATTCTAGTGTGCGTAATGAGTTGTAAAATTATGTATAAACC |
| PGK1p-R-INO2 | AGTTGCTTGTTGCATCATTGTTTTATATTTGTTGTAAAAAGTAGATAATTACTTCCTTG |
| PGK1p-INO2-F | AAATATAAAACAATGATGCAACAAGCAACTGGGAAC |
| INO2-R-TDH2t | CTTAAGGAGTTAAATTCAGGAATCATCCAGTATGTGCTG |
| INO2-TDH2t-F | CTGGATGATTCCTGAATTTAACTCCTTAAGTTACTTTAATGATTTAGTTTTTATTATTAATAATTCATG |
| TDH2t-R-MET17 | AACCCTTGCATCCGAGCGAAAAGCCAATTAGTGTGATACTAAG |
| TDH2t-MET17-F | TAATTGGCTTTTCGCTCGGATGCAAGGGTTCGAATC |
| MET17-R-URA3 | TGTAGAGACCACATCACTGTTCTTTGATGTTAGAACAATTTAGGTTC |
| MET17-URA3-F | ACATCAAAGAACAGTGATGTGGTCTCTACAGGATCTGACA |
| TDH3p-R-HEM3 | AGTTTCAGGGCCCATTTTGTTTGTTTATGTGTGTTTATTCGAAACTAAGT |
| TDH3p-HEM3-F | ACATAAACAAACAAAATGGGCCCTGAAACTCTACATATTG |
| HEM3-R-CYC1t | TAACTAATTACATGATCATTTGATTCTGTCTAAATTAATTTCATCCAGAATTT |
| HEM3-CYC1t-F | GACAGAATCAAATGATCATGTAATTAGTTATGTCACGCTTACATTCA |
| CYC1t-R-MET17 | AACCCTTGCATCCGAGCAAATTAAAGCCTTCGAGCGTC |
| CYC1t-MET17-F | AAGGCTTTAATTTGCTCGGATGCAAGGGTTCGAATC |
| TEF1p-R-POS5 | TGAATCCAACGTACTCCATTTTGTAATTAAAACTTAGATTAGATTGCTATGCT |
| TEF1p-POS5-F | TTAATTACAAAATGGAGTACGTTGGATTCACATTCCCTAAAG |
| POS5-R-TDH2t | CTTAAGGAGTTAAATAGAGAATCTCATTGAATCTTTGCATTCAGA |
| POS5-TDH2t-F | TCAATGAGATTCTCTATTTAACTCCTTAAGTTACTTTAATGATTTAGTTTTTATTATTAATAATTCATG |
| TDH2t-R-PGK1p | ATAATATCTGTGCGTGCGAAAAGCCAATTAGTGTGATACTAAG |
| TDH2t-PGK1p-F | TAATTGGCTTTTCGCACGCACAGATATTATAACATCTGCATAATAGG |
| PGK1p-R-HAC1 | ATCAGTCATTTCCATCATTGTTTTATATTTGTTGTAAAAAGTAGATAATTACTTCCTTG |
| PGK1p-HAC1-F | AAATATAAAACAATGATGGAAATGACTGATTTTGAACTAACTAGTAATTC |
| HAC1-R-TDH2t | CTTAAGGAGTTAAATCTGGATTACGCCAATTGTCAAGATCA |
| HAC1-TDH2t-F | ATTGGCGTAATCCAGATTTAACTCCTTAAGTTACTTTAATGATTTAGTTTTTATTATTAATAATTCATG |

**Table S3.** Synthesized DNA sequences.

| **Name** | sequences |
| --- | --- |
| SmCPS | ATGGCCTCTTTGTCCTCTACCATTTTGTCTAGATCTCCAGCTGCTAGAAGAAGAATTACTCCAGCTTCTGCTAAATTGCATAGACCAGAATGTTTTGCTACTTCTGCTTGGATGGGTTCCTCTTCTAAAAACTTGTCTTTGAGCTACCAGCTGAACCACAAGAAAATTTCTGTTGCTACTGTTGATGCCCCACAAGTTCATGATCACGATGGTACTACTGTTCATCAAGGTCATGATGCCGTTAAGAACATTGAAGATCCAATCGAGTACATCAGAACCTTGTTGAGAACTACTGGTGATGGTAGAATTTCCGTTTCTCCATATGATACTGCTTGGGTTGCTATGATCAAAGATGTTGAAGGTAGAGATGGTCCACAATTCCCATCTTCATTGGAATGGATCGTCCAAAATCAATTGGAAGATGGTTCTTGGGGTGACCAAAAGTTGTTTTGTGTTTACGATAGACTGGTCAACACCATTGCTTGTGTTGTTGCTTTGAGATCTTGGAATGTTCATGCCCATAAGGTTAAGAGAGGTGTCACTTACATCAAAGAAAACGTCGACAAATTGATGGAAGGTAACGAAGAACATATGACCTGTGGTTTTGAAGTTGTCTTTCCAGCTTTGTTGCAAAAGGCTAAGTCTTTGGGTATTGAGGATTTGCCATATGATTCACCAGCTGTTCAAGAAGTTTACCACGTTAGAGAACAGAAGTTGAAGAGAATCCCATTGGAAATCATGCACAAGATCCCTACTAGCTTGTTGTTCTCTTTGGAAGGTTTGGAAAATTTGGACTGGGACAAGTTGTTGAAGTTGCAATCTGCTGATGGTTCCTTTTTGACTTCTCCATCTTCTACTGCTTTCGCCTTTATGCAAACTAAGGACGAAAAGTGCTACCAATTCATCAAGAACACTATCGACACTTTTAATGGTGGTGCTCCACATACTTATCCAGTTGATGTTTTTGGTAGATTGTGGGCCATTGATAGATTGCAAAGATTGGGTATCTCCAGGTTTTTCGAACCAGAAATTGCTGATTGCTTGTCCCATATTCATAAGTTCTGGACTGATAAGGGTGTGTTCTCTGGTAGAGAATCTGAATTCTGCGATATCGATGATACCTCTATGGGTATGAGATTGATGAGAATGCATGGTTACGATGTTGATCCAAACGTCTTGAGAAACTTCAAGCAAAAGGACGGTAAATTCTCTTGTTACGGTGGTCAAATGATCGAATCTCCATCTCCAATCTACAACTTGTACAGAGCTTCCCAATTGAGATTTCCAGGTGAAGAAATCTTGGAGGATGCTAAGAGATTTGCCTACGATTTCTTGAAAGAGAAGTTGGCTAACAACCAGATATTGGATAAGTGGGTTATCTCTAAACACTTGCCAGACGAAATCAAGTTGGGTTTAGAAATGCCATGGTTGGCTACTTTGCCAAGAGTTGAAGCTAAGTACTACATTCAATATTACGCCGGTTCTGGTGATGTTTGGATTGGTAAAACACTGTACAGAATGCCCGAAATCTCTAACGATACTTACCATGATTTGGCCAAGACCGATTTCAAAAGATGCCAAGCTAAACACCAATTCGAGTGGTTGTATATGCAAGAATGGTACGAATCTTGCGGTATCGAAGAATTCGGTATCTCTAGAAAGGACCTGTTGTTGTCTTACTTTTTGGCTACCGCTTCCATTTTCGAATTGGAAAGAACTAACGAAAGAATTGCTTGGGCCAAGTCTCAAATTATTGCCAAGATGATTACCTCGTTCTTCAACAAAGAAACCACTTCCGAAGAAGATAAGAGAGCCTTGTTGAACGAATTGGGTAACATTAACGGTTTGAACGATACAAATGGTGCAGGTAGAGAAGGTGGTGCTGGTTCTATTGCTTTAGCTACTTTGACTCAATTCTTGGAGGGTTTCGATAGATACACCAGACATCAATTGAAGAATGCTTGGTCTGTTTGGTTGACCCAATTGCAACATGGTGAAGCTGATGATGCTGAATTATTGACTAACACCTTGAACATTTGCGCTGGTCATATTGCCTTCAGGGAAGAAATTTTAGCTCACAATGAGTACAAGGCCCTGTCTAACTTGACTTCTAAAATCTGTAGACAGCTGAGCTTCATCCAGTCCGAAAAAGAAATGGGTGTAGAAGGTGAAATTGCCGCCAAATCTTCCATTAAGAACAAAGAACTGGAAGAGGACATGCAGATGTTGGTTAAGTTGGTTTTGGAAAAGTACGGTGGTATCGACAGAAACATTAAGAAGGCTTTTTTGGCTGTTGCCAAGACCTATTACTATAGAGCTTATCATGCTGCCGATACCATTGATACCCATATGTTTAAGGTTTTGTTTGAGCCAGTTGCCTAA |
| SmKSL | ATGTCCTTGGCTTTTAATCCAGCTGCTACTGCTTTTTCTGGTAATGGTGCTAGATCCAGAAGAGAAAATTTCCCAGTCAAACACGTTACCGTTAGAGGTTTTCCAATGATCACTAACAAGTCATCCTTCGCTGTTAAGTGTAACTTGACTACTACTGACTTGATGGGTAAGATTGCCGAAAAGTTTAAGGGCGAAGATTCTAATTTTCCAGCTGCCGCTGCTGTTCAACCAGCTGCTGATATGCCATCTAACTTGTGTATTATCGACACCTTGCAAAGATTGGGTGTTGACAGATACTTCAGATCCGAAATTGATACCATCTTGGAAGATACCTACAGATTGTGGCAGAGAAAAGAAAGAGCCATTTTCTCTGATACCGCTATTCATGCTATGGCTTTTAGACTGTTGAGAGTCAAAGGTTACGAAGTCTCTTCTGAAGAATTGGCTCCATATGCTGATCAAGAACATGTTGACTTGCAAACCATTGAAGTTGCTACCGTTATTGAGTTGTATAGAGCTGCTCAAGAAAGAACCGGTGAAGATGAATCTTCCTTGAAAAAATTGCATGCTTGGACTACCACCTTCTTGAAGCAAAAGTTGTTGACCAATTCCATTCCAGACAAGAAGTTGCATAAGTTGGTTGAGTACTACCTGAAGAACTACCACGGTATTTTGGATAGAATGGGTGTCAGACAAAACTTGGACTTGTACGATATCTCTTACTACAGAACTTCTAAGGCTGCCAACAGATTCTCTAATTTGTGCTCTGAAGATTTCTTGGCTTTCGCTAGACAAGATTTCAACATTTGCCAAGCTCAACACCAGAAAGAATTGCAACAATTGCAGAGATGGTACGCTGATTGCAAATTGGATACTTTGAAGTACGGTAGAGATGTTGTTAGAGTCGCTAACTTTTTGACCTCCGCTATTATTGGTGATCCAGAATTGTCTGATGTCAGAATCGTTTTCGCTCAACACATCGTTTTGGTTACCAGAATCGATGATTTCTTCGATCACAGAGGTTCCAGAGAAGAATCCTACAAGATTTTGGAGTTGATCAAAGAGTGGAAAGAAAAACCAGCAGCTGAATACGGTTCAGAAGAAGTCGAAATTTTGTTCACCGCTGTTTACAACACCGTTAACGAATTGGCTGAAAGGGCTCATGTTGAACAAGGTAGATCTGTTAAGGACTTCCTGATTAAGTTGTGGGTTCAGATCTTGTCCATCTTCAAGAGAGAATTAGATACCTGGTCTGATGATACTGCTTTGACCTTGGATGATTACTTGTCTGCTTCTTGGGTTTCTATTGGTTGCAGAATCTGCATCTTGATGTCCATGCAATTCATCGGTATCAAGTTGTCTGACGAGATGTTGCTATCCGAAGAATGTATTGATTTGTGCAGGCACGTTAGTATGGTTGATAGGTTGTTGAACGATGTCCAGACCTTTGAAAAAGAGCGTAAAGAAAACACCGGTAACTCTGTTACTTTGTTGTTGGCTGCTAACAAGGACGATTCTTCATTCACAGAAGAAGAAGCCATTAGGATCGCTAAAGAAATGGCTGAATGTAACAGACGTCAATTGATGCAAATCGTTTACAAGACCGGTACTATCTTCCCAAGACAATGTAAGGACATGTTTTTGAAGGTCTGCAGAATTGGTTGTTACTTGTACGCTTCTGGTGATGAATTCACTTCTCCACAACAAATGATGGAAGATATGAAGTCCTTGGTCTACGAACCATTGACTATTCATCCATTGGTTGCCAACAATGTCAGAGGTAAGTAA |
| mCherry | ATGGTGAGCAAGGGCGAGGAGGATAACATGGCCATCATCAAGGAGTTCATGCGCTTCAAGGTGCACATGGAGGGCTCCGTGAACGGCCACGAGTTCGAGATCGAGGGCGAGGGCGAGGGCCGCCCCTACGAGGGCACCCAGACCGCCAAGCTGAAGGTGACCAAGGGTGGCCCCCTGCCCTTCGCCTGGGACATCCTGTCCCCTCAGTTCATGTACGGCTCCAAGGCCTACGTGAAGCACCCCGCCGACATCCCCGACTACTTGAAGCTGTCCTTCCCCGAGGGCTTCAAGTGGGAGCGCGTGATGAACTTCGAGGACGGCGGCGTGGTGACCGTGACCCAGGACTCCTCCCTGCAGGACGGCGAGTTCATCTACAAGGTGAAGCTGCGCGGCACCAACTTCCCCTCCGACGGCCCCGTAATGCAGAAGAAGACCATGGGCTGGGAGGCCTCCTCCGAGCGGATGTACCCCGAGGACGGCGCCCTGAAGGGCGAGATCAAGCAGAGGCTGAAGCTGAAGGACGGCGGCCACTACGACGCTGAGGTCAAGACCACCTACAAGGCCAAGAAGCCCGTGCAGCTGCCCGGCGCCTACAACGTCAACATCAAGTTGGACATCACCTCCCACAACGAGGACTACACCATCGTGGAACAGTACGAACGCGCCGAGGGCCGCCACTCCACCGGCGGCATGGACGAGCTGTACAAGTAG |
| CYP76AH24 | ATGTTGCAATTGGGTTCTCAACCACACGAAACTTTTGCTAAGCTGTCTAAAAAGTACGGCCCATTGATGTCTATTCACTTGGGTTCATTATACACCGTCATCGTTTCTTCACCAGAAATGGCCAAAGAAATCATGCATAAGTACGGTCAAGTTTTCTCCGGTAGAACTATTGCTCAAGCAGTTCATGCTTGTGATCACGATAAGATTTCTATGGGTTTTTTGCCAGTTGGTGCTGAATGGCGTGATATGAGAAAAATCTGCAAAGAACAGATGTTCTCCCACCAGTCTATGGAAGATTCACAAAACTTGAGAAAGCAGAAGTTGCAACAGTTGTTGGATTACACCCAAAAGTGTTCTGAAGAAGGTAGAGGTATCGATATTAGAGAAGCTGCTTTCATTACCACCTTGAACTTGATGTCTGCTACCTTGTTTTCTATGCAAGCTACCGAATTCGATTCCAAGGTTACCATGGAATTCAAAGAAATTATCGAAGGTGTTGCCTCCATAGTTGGTGTTCCAAATTTTGCTGATTACTTCCCAATTTTGAGGCCATTTGATCCACAAGGTGTTAAGAGAAGGGCTGATGTTTACTTCGGTAGATTATTGGGTTTGATCGAGGGTTACTTGAACGAAAGGATTGAATTCAGAAAGGCTAATCCAAACGCTCCAAAGAAGGATGATTTTTTGGAAACCTTGGTTGATGCTTTGGATGCCAAAGACTACAAGTTGAAAACTGAACATTTGACCCACCTGATGTTGGATTTGTTTGTTGGTGGTTCTGAAACTTCCACCACTGAAATTGAATGGATCATGTGGGAATTAGTTGCCTCTCCAGAAAAAATGGCTAAGGTTAAGGCTGAATTGAAGTCTGTTATGGGTGGTGAAAAGGTTGTTGACGAATCTATGATGCCAAGATTGCCATACTTGCAAGCTGTTGTCAAAGAATCCATGAGATTGCATCCACCAGGTCCTTTGTTGTTGCCAAGAAAAGCTGAATCTGATCAAGTTGTCAACGGCTACTTGATTCCAAAAGGTACACAAGTTTTGATTAACGCTTGGGCTATGGGTAGAGATTCTTCTTTGTGGAAAAACCCAGATTCCTTCGAACCAGAAAGATTCTTGGATCAAAAGATCGACTTCAAGGGTACTGACTACGAATTGATTCCTTTTGGTTCTGGTAGAAGAGTTTGTCCAGGTATGCCATTGGCTAATAGAATCTTGCATACTGTTACCGCCACTTTGGTTCATAATTTCGATTGGAAATTGGAAAGACCAGAAGCTAACGACGCTCATAAGGGTGTTTTGTTTGGTTTTGCTGTTAGAAGGGCAGTTCCATTGAAAATCGTTCCAATCAAGGCTTAA |
| CYP76AK6 | ATGCAGGTCTTGATCTTGTTGTCTTTGGCTTTTTTGGCTTCTTGCGTTGTTGCTTATTCTAGACGTAGACCAGGTGGTAGAGGTGCTGGTGATTTGCCACCAGGTCCACCAAGATTGCCAATTATTGGTAATATGTTGCAGTTGGGTCAAAACCCACATAAGTCTTTAGCTCATTTGGCTAAAACTTACGGCCCATTGATGTCTTTGAAGTTGGGTAATCAATTCGTCGTCGTTGTTTCTTCACCAGAAATGGCTAGAGAAGTCTTGCAAAGACATGGTTTGGTTTTCTCAAGACCATTCACTCCAATTGCCGTTCAAATTTTAGGTCACGGTGAAGTCTCTATGAACATGTTGCCAGCTACTTCTCCAATTTGGAAGAAGATTAGAAAGATCGCCAGGGAAAAGTTGTTCTCTAATCAAGCTTTACATGCTACCAGAGCCGTTAGAAGAGAAAGATTGAGAAAATTGGCTGACTACGTTGGTAGATGTTCTGGTGCTATGAATGTTGGTGAAGCTACTTTCACTACCATGTCCAATTTGATGTTCGCTACCTTGTTCTCCGTTGAAATTACTCAATACGCCGATTCTGATTCCGATTCTGGTGTTAACAAAAAGTTCAGAGAACACGTTAACGCCATTACTAGATATATGGGTGTTCCAAACATTGCCGATTTCTTCCCAATTTTTGCTCCATTTGATCCACAGGGTTTGAGAAGAAAATTGACCTATCATCTGGGTTCCTTGTTGGAATTGGTTCAGTCTTTGATTGAACAGAGATTGAGAGCTAGAAACGCTGCTACTTACAGAAAGAAGGATGACTTCTTGGAAATGCTGTTGGATTTGTCTGAAGGTGATGAATACGACTTGTCCGTCAACGAAATCAAACATTTGTGCGTCGATTTGATTATCGCTGGTTCTGATACTTCTGCTGCTACTACTGAATGGGCTATGGTTGAATTGCTATTGCATCCAGATAAGTTGGCTAAGTTGAAGGCTGAATTGAAGTCTGTTGTTGGTGACAAGTCCATCATCGAAGAATCCGATATTTCTAAGTTGCCATACTTGCAAGCTACCGTCAAAGAAGTTTTGAGATATCATCCAGCTGCTCCATTATTGGCTCCACATTTGGCTGAAGAAGAAACTCAATTGAACGGTTACATCATCCCAAAGAACACTAAGATCTTCATCAACGATTGGACCATCTCAAGAGATCCATCTATTTGGAAAAACCCAGAAATGTTCGAACCCGAAAGATTCTTGAACAACGATATTGATTTCTGCGGTCAGCACTTTGAATTGATTCCATTTGGTTCCGGTAGAAGAATTTGTCCAGGTTTGCCATTGGCTTCTAGAATGTTGCATTGCATGGTTGCTACTTTGTGCCATAATTTCGACTGGGAATTAGAAAAGGGTACTGAGTCTAAACAATTGCAAAGAGAGGACGTTTTTGGTTTGGCCTTGCAAAAAAAGATTCCATTGAGAGCCATTCCAATCAAGGTCTGA |
| CYP76AH1 | ATGGATTCTTTTCCATTGTTGGCTGCTTTGTTTTTTATTGCTGCTACTATTACTTTTTTGTCTTTTAGAAGAAGAAGAAATTTGCCGCCTGGTCCATTTCCATATCCAATTGTTGGTAATATGTTGCAATTGGGTGCTAATCCACATCAAGTTTTTGCTAAATTGTCTAAAAGATATGGTCCATTGATGTCTATTCATTTGGGTTCTTTGTATACTGTTATTGTTTCTTCTCCTGAAATGGCTAAAGAAATTTTGCATAGACATGGTCAAGTTTTTTCTGGTAGAACTATTGCTCAAGCTGTTCATGCTTGTGATCATGATAAAATTTCTATGGGTTTTTTGCCTGTTGCTTCTGAATGGAGAGATATGAGAAAAATTTGTAAAGAACAAATGTTTTCTAATCAATCTATGGAAGCTTCTCAAGGTTTGAGAAGACAAAAATTGCAACAATTGTTGGATCATGTTCAAAAATGTTCTGATTCTGGTAGAGCTGTTGATATTAGAGAAGCTGCTTTTATTACTACTTTGAATTTGATGTCTGCTACTTTGTTTTCTTCTCAAGCTACGGAATTCGACTCTAAAGCTACTATGGAATTCAAAGAGATTATTGAAGGTGTTGCTACTATTGTTGGTGTTCCAAATTTTGCTGATTATTTTCCAATTTTGAGACCATTTGATCCACAAGGCGTTAAGAGGAGAGCTGATGTTTTTTTTGGTAAGTTGTTGGCGAAGATTGAGGGTTACCTTAATGAAAGATTGGAATCTAAAAGAGCGAACCCAAATGCACCTAAGAAAGACGATTTTCTCGAGATAGTGGTTGATATTATTCAAGCCAACGAATTCAAGTTAAAGACTCACCATTTTACTCATTTGATGTTGGATTTGTTTGTTGGTGGTTCTGATACTAATACTACTTCTATTGAATGGGCTATGTCTGAATTGGTTATGAATCCTGATAAAATGGCTAGATTGAAAGCTGAATTGAAATCTGTTGCTGGTGATGAAAAAATTGTTGATGAATCTGCTATGCCAAAATTGCCATATTTGCAAGCTGTTATTAAAGAAGTTATGAGAATTCATCCTCCTGGTCCATTGTTGTTGCCAAGAAAAGCTGAATCTGATCAAGAAGTTAATGGTTATTTGATTCCAAAAGGTACTCAAATTTTGATTAATGCTTATGCTATTGGTAGAGATCCATCTATTTGGACTGACCCGGAGACTTTTGATCCTGAACGCTTTTTAGATAACAAAATTGATTTTAAGGGTCAAGATTATGAATTGTTGCCATTTGGTTCTGGTAGAAGAGTTTGTCCTGGTATGCCATTGGCTACTAGAATTTTGCATATGGCTACTGCTACTTTGGTTCATAATTTTGATTGGAAATTGGAAGATGATTCTACTGCTGCTGCTGATCATGCTGGTGAATTGTTTGGTGTTGCTGTTAGAAGAGCTGTTCCATTGAGAATTATTCCAATTGTTAAATCTTAA |
| SmCPR | ATGGAACCATCTTCTAAAAAATTGTCTCCATTGGATTTTATTACTGCTATTTTGAAAGGTGATATTGAAGGTGTTGCTCCAAGAGGTGTTGCTGCTATGTTGATGGAAAATAGAGATTTGGCTATGGTTTTGACTACTTCTGTTGCTGTTTTGATTGGTTGTGTTGTTGTTTTGGCTTGGAGAAGAACTGCTGGTTCTGCTGGTAAAAAACAATTGCAACCACCAAAATTGGTTGTTCCAAAACCTGCTGCTGAACCTGAAGAAGCTGAAGATGAAAAAACTAAAGTTTCTGTCTTTTTTGGTACTCAAACTGGTACTGCTGAAGGTTTTGCTAAAGCTTTTGCTGAAGAAGCTAAAGCTAGATATCCACAAGCTAAATTTAAAGTTATTGATTTGGATGATTATGCTGCTGATGATGATGAGTATGAAGAAAAATTGAAAAAGGAATCTTTGGCTTTTTTCTTTTTGGCTTCTTATGGTGATGGTGAACCAACTGATAATGCTGCTAGATTTTATAAATGGTTTACTGAAGGTAAAGATAGAGAAGATTGGTTGAAAAATTTGCAGTATGGTGTTTTTGGTTTGGGTAATAGACAGTATGAACACTTCAACAAGATTGCTATTGTTGTTGATGATTTAATTACTGAACAAGGTGGTAAAAAATTGGTTCCTGTTGGTTTGGGTGATGATGATCAATGTATTGAAGATGATTTTTCTGCTTGGAGAGAATTGGTTTGGCCTGAATTGGATAAATTGTTGAGAAATGAAGATGATGCTACTGTTGCTACTCCATATACTGCTGTTGTTTTGCAATATAGAGTTGTTTTGCATGATCAAACTGATGGTCTGATTACTGAAAATGGTTCTCCAAATGGTCATGCTAATGGTAATACTATTTATGATGCTCAACATCCATGTAGAGCTAATGTTGCTGTTAGAAGAGAATTGCATACTCCTGCTTCTGATAGATCTTGTACTCATTTGGAATTTGATACTTCTGGTACTGGTTTGGTTTATGAAACTGGTGATCATGTTGGTGTTTATTGTGAAAATTTGTTGGAAAATGTTGAAGAAGCTGAAAAGCTTTTGAATTTGTCTCCACAAACTTATTTTTCTGTTCATACTGATAATGAAGATGGTACTCCATTGTCTGGTTCTTCTTTGCCACCACCATTTCCACCATGTACTTTGAGAACTGCTTTGACTAAATATGCTGATTTGATTTCTATGCCTAAAAAATCTGTTTTGGTTGCTTTGGCTGAATATGCTTCTAATCAATCTGAAGCTGATAGATTGAGATATTTGGCTTCTCCTGATGGTAAAGAAGAATATGCTCAATATATTGTTGCTTCTCAAAGATCTTTGTTGGAAGTTATGGCTGAATTTCCATCTGCTAAACCACCATTGGGTGTCTTTTTTGCTGCTATTGCTCCAAGATTGCAACCAAGATTTTATTCTATTTCTTCTTCTCCAAAAATTGCTCCAACTAGAGTTCATGTTACTTGTGCTCTAGTGTATGACAAAACTCCAACTGGTAGAATTCATAAAGGTATTTGTTCTACTTGGATTAAAAATGCTGTTCCATTGGAAGAATCTTCTGATTGTTCTTGGGCTCCAATTTTTATTAGAAATTCTAATTTTAAATTGCCTGCTGATCCAAAAGTTCCAATTATTATGGTTGGTCCTGGTACTGGTTTGGCTCCATTTAGAGGTTTTTTGCAAGAAAGATTGGCTTTGAAAGAATCTGGTGCTGAATTGGGTCCTGCTATCTTGTTCTTTGGCTGTAGAAATAGAAAGATGGACTTTATTTATGAAGATGAACTCAATTCTTTTGTTAAAGTTGGTGCTATTTCTGAATTGATTGTTGCTTTTTCTAGAGAAGGTCCTGCTAAAGAATATGTTCAACATAAAATGTCTCAAAGAGCTTCTGATATTTGGAAAATGATTTCTGATGGTGGTTATATGTATGTTTGTGGTGATGCTAAAGGTATGGCTAGAGATGTTCATAGAACTTTGCATACTATTGCTCAAGAACAAGGTTCTTTGTCTTCTTCTGAAGCTGAAGGTATGGTTAAGAATTTGCAAACTACTGGTAGATATTTGAGAGATGTTTGG |
| SpCyb5 | ATGGCAAAATCTCATACTTTTGAAGAAGTTGCAAAACATAACAAGACTAAAGATTGTTGGTTGATTATTTCTGGTAAAGTTTATGATGTTACTCCATTTATGGAAGATCATCCTGGTGGTGATGAAGTTTTGTTGTCTGCTACTGGTAAAGATGCTACTAATGATTTTGAAGATGTTGGTCATTCTGATTCTGCTAGAGAAATGATGGATAAATATTTTATTGGTGAAATTGATATGGCTACTGTTCCATTGAAAAGATCTTATATTGCTCCACAACAACCATCTTATAATCCTGATAAAACTCCTGAATTTGTTATTAAAATTTTGCAATTTTTGGTTCCATTGTTGATTTTGGGTTTGGCTTTTGCTGTTAGATTGTATACTAAAGAAAAATAA |
| GuCPR | ATGACCTCCAACTCTGATTTGGTTAGAACCATCGAATCTGTTTTGGGTGTTTCTTTGGGTGATTCCGTTTCTGATTCCTTGGTTTTGATTGCTACTACCTCCGTTGCTGTTATTATCGGTTTGTTGGTTTTCCTGTGGAAGAAGTCATCTGACAGATCTAGAGAAGTTAGACCAGTTATCGTTCCAAAGTCCTTGGTTAAGGATGAAGATGATGATGTTGATGTCGCTTCTGGTAAGACTAAGGTTACTGTTTTTTTCGGTACTCAAACCGGTACTGCTGAAGGTTTTGCTAAAGCTTTGGCTGACGAAATCAAAGCCAGATACGAAAAAGCTTACGTTAAGGTTGTTGACTTGGATGATTACGCCATGGATGATGATCAATACGAAGAGAAGTTGAAGAAAGAAACCTTGGCCTTTTTCATGTTGGCTACTTATGGTGATGGTGAACCTACTGATAATGCTGCTAGATTTTACAAGTGGTTCACCGAAGGTAAAGAAGAAAGAGGTACTTGGTTGCAACAATTGACTCATGGTGTTTTTGGTTTGGGTAACAAGCAATACGAACACTTCAACAAGATTGGTAAGGTTGTCGACGAAGATTTGTCTGAACAAGGTGCTAAAAGATTGGTCCCATTAGGTTTAGGTGATGACGATCAATCTATCGAGGATGATTTTTCCGCTTGGAAAGAATCTTTGTGGCCAGAATTGGACCAACTGTTGAGAGATGAGGATGATGTTAACACTGTTTCTACTCCATACACTGCTGCTATTCCAGAGTACAGAGTTGTTATTCATGATTCCACTGTTACCCCATCCTACGACAATCAATTTTCTGCTGCTAATGGTGGTGCCGTTTTCGATATTCATCATCCTTGTAGAGTTAACGTTGCCGTCAAAAGAGAATTGCATAAGCCACAATCTGATAGGTCCTGCATTCATTTGGAATTCGATATTTCCGGTACTGGTATTACTTACGAAACCGGTGATCATGTTGGTGTTTACGCTGAAAATTGTGACGAAACTGTTGAAGAAGCTGGTAAGTTGTTGGGTCAAAACTTGGATTTGTTGTTCTCCTTGCATACCGATAACGAAGATGGTACTTCTTTAGGTGGTTCTTTGTTGCCACCATTTCCAGGTCCATGTACTTTGAGAACTGCTTTGGCTAGATATGCCGATTTGTTGAATCCACCAAGAAAAGCTGCTTTGGTTGTTTTGGCTGCTCATGCTTCTGAACCATCTGAAGCTGAAAGATTGAAGTTCTTGTCATCTCCACAGGGTAAAGACGAATACTCTAAATGGGTTGTTGGTTCCCAAAGGTCTTTGTTGGAAGTTATGGCTGAATTTCCATCTGCTAAACCACCATTGGGTGTATTTTTTGCTGCAATTGCTCCAAGATTGCAACCTAGGTATTACTCCATTTCTTCCAGTCCAAGATTTGCCTCTCAAAGGGTTCATGTTACTTGTGCTTTAGTTTATGGTCCAACTCCAACTGGTAGAATCCATAAGGGTGTTTGTTCTACCTGGATGAAGAATGCTATACCATTGGAAGAATCTAGAGATTGTGGTTGGGCTCCAATTTTCATTAGACCATCTAATTTCAAGTTGCCAGCCGATCATTCCATTCCAATTATCATGGTTGGTCCAGGTACTGGTTTGGCTCCTTTTAGAGGTTTTCTGCAAGAAAGATTCGCCTTGAAAGAAGATGGCGTTCAATTGGGTCCATCCTTGTTGTTTTTTGGTTGCAGAAACAGACAGATGGACTTCATATACGAGGACGAGTTGAAAAACTTTGTCGAACAGGGTTCATTGTCCGAATTGATCGTTGCTTTTTCAAGAGAAGGTCCCGAAAAAGAATACGTCCAACATAAGATGATGGATAAGGCTGCTTATCTGTGGTCCTTGATTTCTCAAGGTGGTTACTTGTATGTTTGCGGTGATGCTAAAGGTATGGCTAGAGATGTTCATAGAATCTTGCACACCATCGTCCAACAACAAGAAAACGTTGAATCTTCTAAGGCTGAAGCCATCGTCAAAAAGTTGCAAATGGATGGTAGATACTTGAGGGATGTTTGGTGA |
| AtCPR | ATGACTTCTGCATTATACGCATCAGACTTATTTAAGCAGTTGAAATCTATAATGGGAACAGACTCATTGTCAGACGACGTCGTTTTAGTTATTGCTACTACTTCATTGGCTTTGGTTGCTGGATTTGTTGTTTTATTGTGGAAAAAGACAACAGCTGATAGGTCTGGTGAATTAAAGCCATTAATGATACCTAAATCTTTAATGGCTAAGGACGAGGACGACGACTTGGATTTAGGATCAGGAAAGACTAGAGTCTCTATATTTTTCGGAACTCAGACAGGAACAGCTGAGGGATTCGCAAAGGCTTTATCAGAAGAGATTAAAGCAAGGTACGAGAAGGCTGCTGTCAAAGTTATAGATTTGGATGACTACGCAGCTGATGACGACCAGTACGAGGAAAAGTTGAAAAAGGAAACTTTGGCATTTTTCTGTGTTGCAACATACGGTGACGGTGAGCCAACTGACAACGCTGCTAGGTTCTACAAATGGTTCACAGAGGAAAATGAGAGAGACATTAAATTGCAGCAGTTGGCTTACGGTGTCTTCGCATTGGGAAACAGGCAATATGAACATTTCAATAAGATTGGAATTGTCTTGGACGAAGAATTATGCAAAAAAGGAGCTAAGAGGTTGATAGAGGTCGGTTTGGGTGACGATGACCAGTCAATAGAGGACGACTTCAATGCATGGAAAGAGTCATTGTGGTCAGAGTTAGATAAGTTATTAAAAGACGAAGACGACAAGTCAGTCGCAACACCTTACACAGCAGTCATACCTGAGTATAGGGTCGTCACTCACGACCCAAGATTCACTACTCAAAAGTCAATGGAGTCAAATGTCGCAAACGGAAATACTACTATTGACATTCATCACCCATGCAGGGTTGACGTCGCTGTCCAGAAAGAGTTACACACTCACGAGTCTGACAGGTCATGCATTCACTTGGAGTTCGATATTTCAAGAACTGGTATTACTTACGAAACAGGTGACCACGTTGGTGTCTACGCTGAGAACCACGTCGAGATTGTCGAGGAAGCTGGAAAGTTGTTGGGACATTCTTTAGATTTGGTCTTCTCAATTCATGCTGACAAAGAGGACGGTTCACCATTGGAGTCTGCTGTTCCACCACCATTCCCTGGACCATGCACTTTAGGTACTGGTTTGGCAAGGTACGCAGACTTATTGAACCCACCTAGGAAGTCAGCTTTAGTTGCATTGGCTGCATATGCAACAGAACCATCTGAGGCAGAGAAATTAAAGCACTTGACTTCTCCTGACGGTAAGGACGAGTACTCACAGTGGATAGTCGCATCTCAGAGGTCATTGTTGGAGGTCATGGCAGCATTTCCATCAGCAAAGCCACCTTTAGGTGTTTTCTTCGCAGCTATAGCACCTAGATTGCAGCCTAGGTATTATTCAATATCTTCTTCACCTAGGTTGGCTCCATCTAGGGTCCACGTCACATCAGCTTTGGTTTACGGACCTACTCCTACAGGAAGGATACATAAAGGAGTCTGCTCTACTTGGATGAAGAACGCTGTCCCAGCAGAGAAGTCTCATGAGTGCTCAGGAGCTCCTATTTTTATTAGGGCATCAAATTTCAAATTGCCTTCAAACCCATCTACTCCAATAGTCATGGTCGGACCAGGAACAGGTTTGGCTCCTTTCAGGGGATTTTTGCAGGAGAGGATGGCTTTGAAGGAGGATGGTGAGGAATTGGGATCATCTTTGTTGTTCTTTGGTTGTAGGAATAGGCAAATGGACTTCATTTATGAGGACGAATTGAACAACTTTGTTGATCAAGGAGTCATATCAGAGTTAATTATGGCTTTCTCAAGGGAGGGTGCACAAAAGGAATACGTCCAACACAAGATGATGGAAAAGGCTGCACAGGTCTGGGACTTGATTAAGGAGGAGGGATACTTATATGTCTGCGGTGACGCAAAGGGTATGGCAAGAGACGTCCACAGGACTTTGCACACAATTGTCCAGGAACAGGAGGGTGTTTCTTCATCTGAAGCAGAGGCTATTGTTAAAAAGTTGCAAACTGAAGGTAGGTACTTGAGGGACGTCTGGTAA |
| ScCTT1 | ATGAACGTGTTCGGTAAAAAAGAAGAAAAGCAAGAAAAAAGTTTACTCTCTACAAAACGGTTTTCCGTACTCTCATCACCCATACGCTTCTCAATACTCAAGACCAGACGGCCCTATCTTACTGCAAGACTTCCATCTGCTGGAAAATATGCAAGTTTCGATAGAGAAAGAGTTCCGGAGCGTGTAGTCCATGCCAAAGGTGGTGGTTGTAGACTGGAGTTCGAACTAACAGATTCTTTGAGTGATATTACATACGCCGCTCCATACCAGAATGTGGGTTACAAATGTCCTGGTCTTGTTCGTTTTTCCACCGTTGGTGGTGAAAGTGGTACACCAGACACTGCAAGAGACCCAAGAGGTGTTTCTTTTAAATTCTATACCGAGTGGGGGAACCATGACTGGGTCTTCAACAATACTCCCGTCTTCTTCCTCAGAGACGCTATTAAGTTTCCCGTATTTATTCATTCGCAAAAGAGAGACCCTCAGTCTCATCTGAATCAGTTTCAGGACACTACCATATACTGGGATTATCTAACATTGAATCCGGAATCAATCCATCAAATAACTTACATGTTTGGTGATAGAGGTACTCCTGCTTCGTGGGCTAGTATGAACGCGTACTCTGGTCATTCCTTCATCATGGTCAACAAAGAAGGTAAGGACATATGTGCAATTCCACGTCTTGTCGGATACTGGTTTTGAAACCTTGACTGGAGATAAGGCTGCTGAACTGTCAGGCTCCCACCCTGATTATAATCAGGCAAAGCTGTTCACTCAATTGCAAAATGGCGAAAAGCCAAAATTTAACTGTTATGTGCAAACAATGACACCCGAACAAGCAACTAAGTTCAGGTATTCGGTAAATGACCTAACGAAAATATGGCCACACAAGGAATTCCCTTTGAGAAAATTTGGTACCATCACCCTAACGGAGAATGTTGACAATTATTTCCAAGAAATTGAAACAAGTTGCATTCAGTCCAACGAACACTTGTATCCCAGGTATTAAGCCTTCTAATGATTCCGTTCTACAAGCCAGACTTTTCTCCTATCCAGACACTCAACGTCATAGATTGGGAGCCAACTATCAGCAATTGCCCGTCAACAGACCAAGAAACTTGGGATGTCCATACTCCAAAGGTGATTCCCAATACACTGCCGAACAGTGTCCATTTAAAGCAGTGAACTTCCAAAGGGACGGCCCAATGAGTTACTACAATTTCGGTCCTGAGCCAAATTATATTTCCAGTTTACCAAATCAAACTCTGAAATTCAAAAATGAAGACAACGACGAAGTATCTGATAAGTTCAAAGGGATAGTTCTTTGACGAAGTAACAGAAGTTTCTGTGAGAAAACAGGAACAAGACCAAATCAGAAACGAGCATATTGTTGATGCCAAAATTAATCAATATTACTACGTTTATGGTATTAGTCCACTAGACTTCGAACAGCCAAGAGCTCTATATGAAAAGGTATACAACGATGAACAGAAGAAATTATTCGTTCATAACGTTGTTTGCCACGCTTGTAAGATCAAAGATCCTAAAGTCAAAAAGAGAGTTACGCAATACTTTGGTTTGCTAAACGAAGATTTGGGTAAAGTCATTGCAGAATGCTTGGGAGTTCCTTGGGAACCTGTTGACCTTGAAGGTTATGCCAAGACTTGGTCCATTGCAAGTGCCAATTAA |
| ScCTA1 | ATGTCGAAATTGGGACAAGAAAAAAATGAAGTAAATTACTCTGATGTAAGAGAGGATAGAGTTGTGACAAACTCCACTGGTAATCCAATCAATGAACCATTTGTCACCCAACGTATTGGGGAACATGGCCCTTTGCTTTTGCAAGATTATAACTTAATTGATTCTTTTGGCTCATTTCAACAGGGAAAATATTCCTCAAAGGAATCCACATGCTCATGGTTCTGGTGCCTTCGGCTATTTTGAAGTAACCGATGACATTACTGATATCTGCGGGTCTGCTATGTTTAGTAAAATTGGGAAAAGAACGAAAATGTCTAACAAGATTTTCGACTGTGGGTGGTGATAAAGGTAGTGCCGACACGGTTCGTGATCCAAGGGGGTTTGCCACCAAATTCTACACTGAAGAAGGTAATTTAGATTGGGTCTACAATAATACACCGGTATTCTTTATCAGAGACCCTTCCAAGTTCCCTCACTTTATCCACACACAGAAGAGAAACCCACAAACCAACCTAAGGGATGCTGACATGTTTTGGGATTTCCTCACCACTCCTGAAAATCAGGTGGCCATTCATCAAGTAATGATCCTTTTTTCAGACCGTGGTACCCCTGCCAACTACCGTAGTATGCATGGTTATTCTGGTCATACCTATAAAATGGTCCAATAAAAACGGAGATTGGCATTATGCAAGTTCATATCAAAACCGATCAAGGAATAAAGAATTTGACCATAGAAGAGGCTACCAAAATTGCGGGATCCAATCCAGATTACTGCCAGCAGGATTTATTTGAGGCTATTCAGAATGGAAACTATCCTTCCTGGACAGTTTATATTCAAACAATGACCGAACGCGATGCCAAAAAATTACCATTTTCAGTCTTTGATTTGACTAAAGTATGGCCTCAGGGGCAATTCCCTTTACGGCGTGTGGGTAAGATTGTTTTGAACGAGAATCCACTGAACTTCTTCGCACAGGTGGAACAAGCTGCCTTCGCCCCCAGTACCACGGTTCCTTACCAAGAAGCAAGCGCTGATCCAGTATTACAGGCCCGTTTGTTTTCATATGCGGATGCTCATAGATACAGGCTAGGTCCTAACTTCCATCAAATACCCGTAAACTGTCCATATGCATCTAAATTTTTCAATCCCGCTATCAGAGATGGACCGATGAATGTTAACGGCAACTTCGGCTCAGAACCTACATATTTGCCAACGATAAATCGTACACGTATATCCAAACAGGACAGACCCATTCAACAACACCAAGAGGTATGGAATGGCCAGCTATCCCTTATCATTGGGCAACATCCCCAGGTGATGTAGATTTCGTGCAAGCAAGAAATCTCTACCGCGTTTTGGGTAAACCTGGACAGCAAAAGAACTTGGCATATAACATCGGCATTCATGTAGAAGGCGCCTGTCCTCAAATACAGCAGCGCGTTTATGATATGTTTGCTCGTGTTGATAAGGGACTATCTGAGGCAATTAAAAAAGTAGCTGAGGCAAAACATGCTTCTGAGCTTTCGAGTAACTCCAAATTTTGA |

The predicted transmembrane region is marked in green.

**Table S4.** Sequences of promoters and terminators

| **Name** | **Sequences** |
| --- | --- |
| Promotor PGK1 | TATTTTAGATTCCTGACTTCAACTCAAGACGCACAGATATTATAACATCTGCACAATAGGCATTTGCAAGAATTACTCGTGAGTAAGGAAAGAGTGAGGAACTATCGCATACCTGCATTTAAAGATGCCGATTTGGGCGCGAATCCTTTATTTTGGCTTCACCCTCATACTATTATCAGGGCCAGAAAAAGGAAGTGTTTCCCTCCTTCTTGAATTGATGTTACCCTCATAAAGCACGTGGCCTCTTATCGAGAAAGAAATTACCGTCGCTCGTGATTTGTTTGCAAAAAGAACAAAACTGAAAAAACCCAGACACGCTCGACTTCCTGTCTTCCTATTGATTGCAGCTTCCAATTTCGTCACACAACAAGGTCCTAGCGACGGCTCACAGGTTTTGTAACAAGCAATCGAAGGTTCTGGAATGGCGGGAAAGGGTTTAGTACCACATGCTATGATGCCCACTGTGATCTCCAGAGCAAAGTTCGTTCGATCGTACTGTTACTCTCTCTCTTTCAAACAGAATTGTCCGAATCGTGTGACAACAACAGCCTGTTCTCACACACTCTTTTCTTCTAACCAAGGGGGTGGTTTAGTTTAGTAGAACCTCGTGAAACTTACATTTACATATATATAAACTTGCATAAATTGGTCAATGCAAGAAATACATATTTGGTCTTTTCTAATTCGTAGTTTTTTCAAGTTCTTAGATGCTTTCTTTTTCTCTTTTTTTACAGATCATCAAGGAAGTAATTATCTACTTTTTACAACAAATATAAAAAC |
| Promotor TDH3 | ATACTAGCGTTGAATGTTAGCGTCAACAACAAGAAGTTTAATGACGCGGAGGCCAAGGCAAAAAGATTCCTTGATTACGTAAGGGAGTTAGAATCATTTTGAATAAAAAACACGCTTTTTCAGTTCGAGTTTATCATTATCAATACTGCCATTTCAAAGAATACGTAAATAATTAATAGTAGTGATTTTCCTAACTTTATTTAGTCAAAAAATTAGCCTTTTAATTCTGCTGTAACCCGTACATGCCCAAAATAGGGGGCGGGTTACACAGAATATATAACATCGTAGGTGTCTGGGTGAACAGTTTATTCCTGGCATCCACTAAATATAATGGAGCCCGCTTTTTAAGCTGGCATCCAGAAAAAAAAAGAATCCCAGCACCAAAATATTGTTTTCTTCACCAACCATCAGTTCATAGGTCCATTCTCTTAGCGCAACTACAGAGAACAGGGGCACAAACAGGCAAAAAACGGGCACAACCTCAATGGAGTGATGCAACCTGCCTGGAGTAAATGATGACACAAGGCAATTGACCCACGCATGTATCTATCTCATTTTCTTACACCTTCTATTACCTTCTGCTCTCTCTGATTTGGAAAAAGCTGAAAAAAAAGGTTGAAACCAGTTCCCTGAAATTATTCCCCTACTTGACTAATAAGTATATAAAGACGGTAGGTATTGATTGTAATTCTGTAAATCTATTTCTTAAACTTCTTAAATTCTACTTTTATAGTTAGTCTTTTTTTTAGTTTTAAAACACCAAGAACTTAGTTTCGAATAAACACACATAAACAAACAAA |
| Promotor TEF1 | AGTGATCCCCCACACACCATAGCTTCAAAATGTTTCTACTCCTTTTTTACTCTTCCAGATTTTCTCGGACTCCGCGCATCGCCGTACCACTTCAAAACACCCAAGCACAGCATACTAAATTTCCCCTCTTTCTTCCTCTAGGGTGTCGTTAATTACCCGTACTAAAGGTTTGGAAAAGAAAAAAGAGACCGCCTCGTTTCTTTTTCTTCGTCGAAAAAGGCAATAAAAATTTTTATCACGTTTCTTTTTCTTGAAAATTTTTTTTTTTGATTTTTTTCTCTTTCGATGACCTCCCATTGATATTTAAGTTAATAAACGGTCTTCAATTTCTCAAGTTTCAGTTTCATTTTTCTTGTTCTATTACAACTTTTTTTACTTCTTGCTCATTAGAAAGAAAGCATAGCAATCTAATCTAAGTTTTAATTACAAA |
| Terminator ADH2 | GCGGATCTCTTATGTCTTTACGATTTATAGTTTTCATTATCAAGTATGCCTATATTAGTATATAGCATCTTTAGATGACAGTGTTCGAAGTTTCACGAATAAAAGATAATATTCTACTTTTTGCTCCCACCGCGTTTGCTAGCACGAGTGAACACCATCCCTCGCCTGTGAGTTGTACCCATTCCTCTAAACTGTAGACATGGTAGCTTCAGCAGTGTTCGTTATGTACGGCATCCTCCAACAAACAGTCGGTTATAGTTTGTCCTGCTCCTCTGAATCGTCTCCCTCGATATTTCTCATTTTCCTTCGCATGCCAGCATTGAAATGATCGAAGTTCAATGATGAAACGGTAATTCTTCTGTCATTTACTCATCTCATCTCATCAAGTTATATAATTCTA |
| Terminator TDH2 | ATTTAACTCCTTAAGTTACTTTAATGATTTAGTTTTTATTATTAATAATTCATGCTCATGACATCTCATATACACGTTTATAAAACTTAAATAGATTGAAAATGTATTAAAGATTCCTCAGGGATTCGATTTTTTTGGAAGTTTTTGTTTTTTTTTCCTTGAGATGCTGTAGTATTTGGGAACAATTATACAATCGAAAGATATATGCTTACATTCGACCGTTTTAGCCGTGATCATTATCCTATAGTAACATAACCTGAAGCATAACTGACACTACTATCATCAATACTTGTCACATGAGAACTCTGTGAATAATTAGGCCACTGAAATTTGATGCCTGAAGGACCGGCATCACGGATTTTCGATAAAGCACTTAGTATCACACTAATTGGCTTTTCGC |
| Terminator CYC1 | TCATGTAATTAGTTATGTCACGCTTACATTCACGCCCTCCCCCCACATCCGCTCTAACCGAAAAGGAAGGAGTTAGACAACCTGAAGTCTAGGTCCCTATTTATTTTTTTATAGTTATGTTAGTATTAAGAACGTTATTTATATTTCAAATTTTTCTTTTTTTTCTGTACAGACGCGTGTACGCATGTAACATTATACTGAAAACCTTGCTTGAGAAGGTTTTGGGACGCTCGAAGGCTTTAATTTGC |

**Table S5** Overview of the production of miltiradiene, ferruginol carnosic acid and carnosol.

| strain | Miltiradiene titer (mg/L) | Ferruginol titer (mg/L) | CA titer (mg/L) | Carnosol titer (mg/L) |
| --- | --- | --- | --- | --- |
| WM1 | 0.18±0.03 | - | - | - |
| WM2 | 1.27±0.09 | - | - | - |
| WM3a | 10.98±0.86 | - | - | - |
| WM3b | 8.57±0.50 | - | - | - |
| WM3c | 6.64±0.41 | - | - | - |
| WM3d | 0.71±0.50 | - | - | - |
| WM3e | 5.63±0.56 | - | - | - |
| WM4 | 172.77±3.67 | - | - | - |
| WCA1a | 172.37±3.33 | - | 0.03±0.002 | - |
| WCA1b | 172.35±3.27 | - | 0.04±0.003 | - |
| WCA1c | 171.98±2.41 | - | 0.05±0.003 | - |
| WCA2 | 168.53±2.70 | 0.97±0.16 | 0.82±0.04 | - |
| WCA3 | 163.78±3.03 | 4.15±0.37 | 2.62±0.37 | - |
| WCA4a | 170.77±2.61 | - | 0.16±0.01 | - |
| WCA4b | 160.34±2.95 | 4.98±0.19 | 2.81±0.19 | - |
| WCA4c | 160.67±2.42 | 5.63±0.15 | 3.17±0.29 | 0.03±0.002 |
| WCA4d | 170.23±3.10 | - | 0.31±0.16 | - |
| WCA5 | 151.25±4.21 | 12.74±1.02 | 4.30±0.72 | 0.08±0.003 |
| WCA6 | 156.10±3.58 | 9.31±0.89 | 6.20±1.38 | 0.13±0.03 |
| WCA7a | 149.19±2.88 | 11.38±2.00 | 7.57±1.52 | 0.23±0.07 |
| WCA7b | 147.65±2.19 | 14.64±1.63 | 8.84±1.67 | 0.45±0.10 |
| WCA7c | 152.32±3.31 | 10.25±1.89 | 6.83±1.59 | 0.12±0.08 |
| WCA8 | 139.76±3.29 | 20.25±1.11 | 11.42±1.54 | 0.76±0.14 |
| WCA9 | 125.98±3.90 | 28.35±1.11 | 17.12±1.74 | 1.90±0.27 |
| WCA10 | 116.76±4.50 | 33.86±1.25 | 20.54±1.61 | 3.24±0.45 |
| WCA11 | 109.89±4.55 | 36.29±1.87 | 24.65±1.42 | 4.90±0.33 |


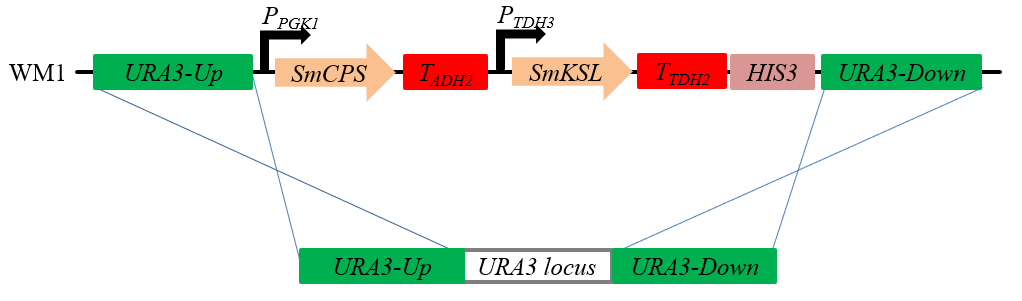

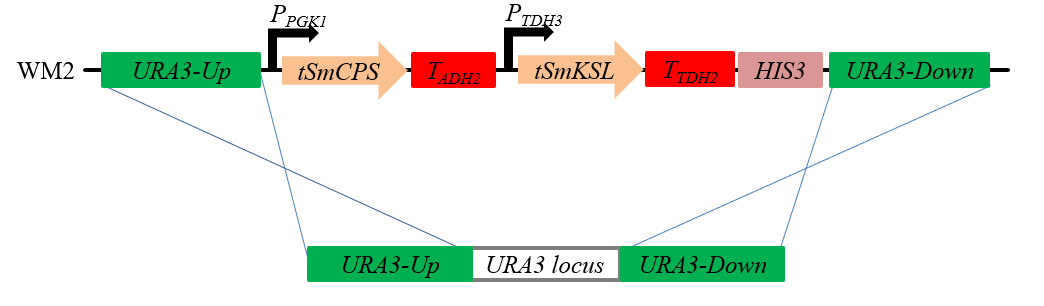


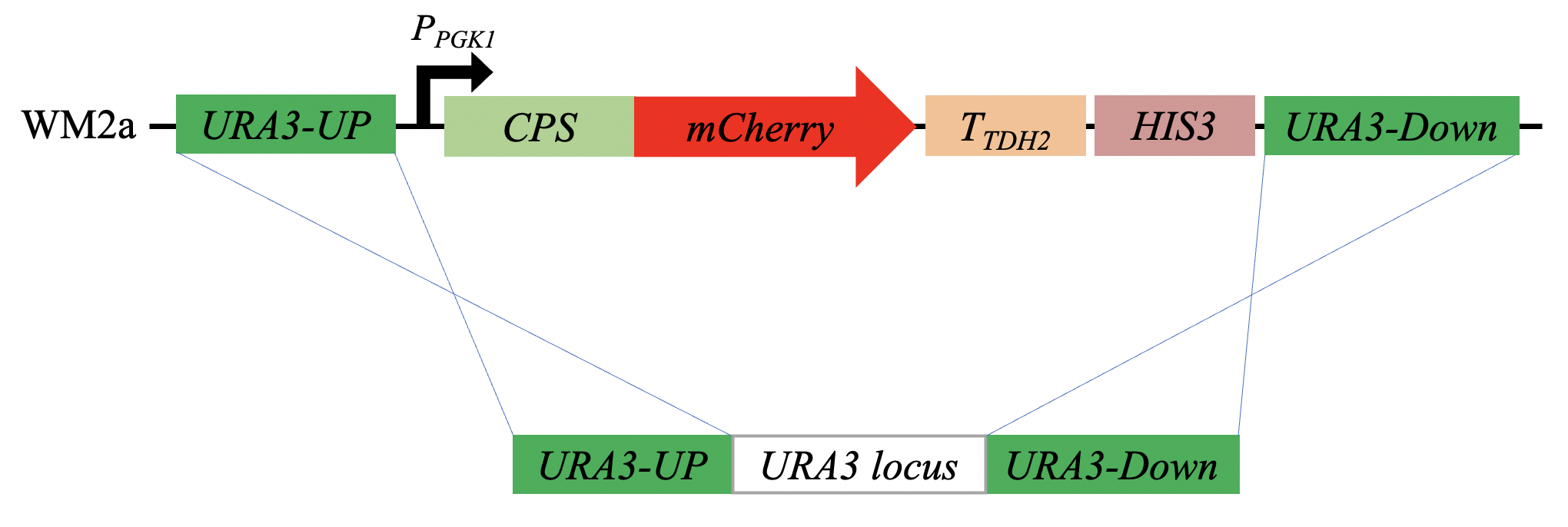

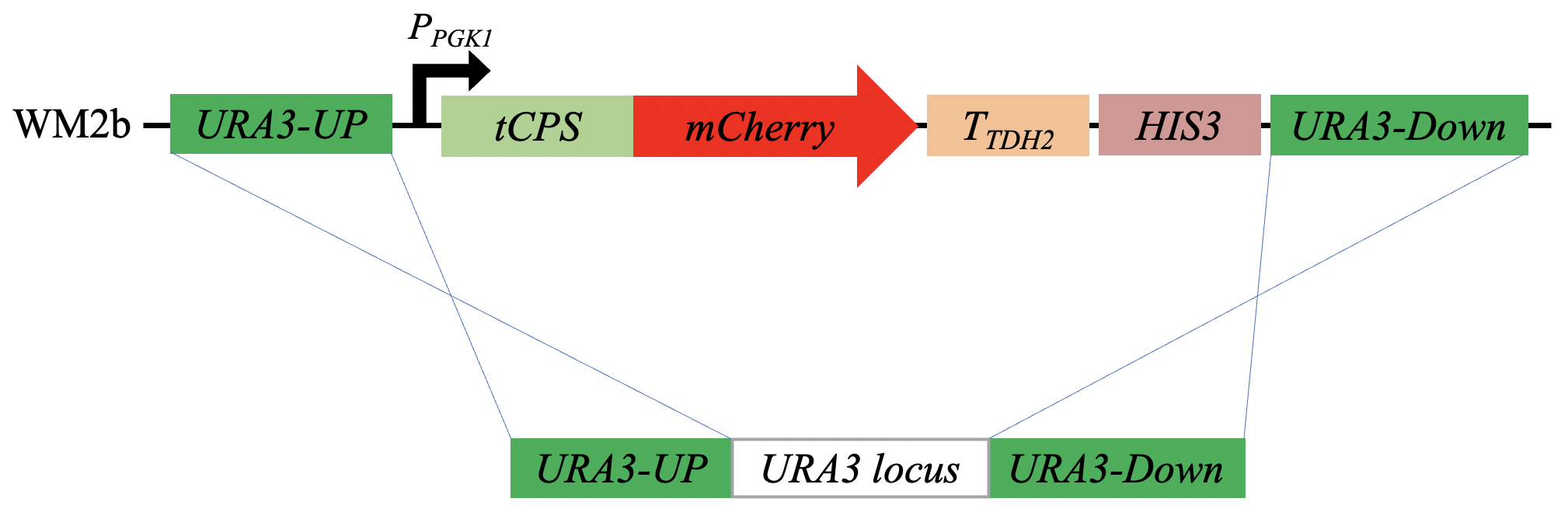

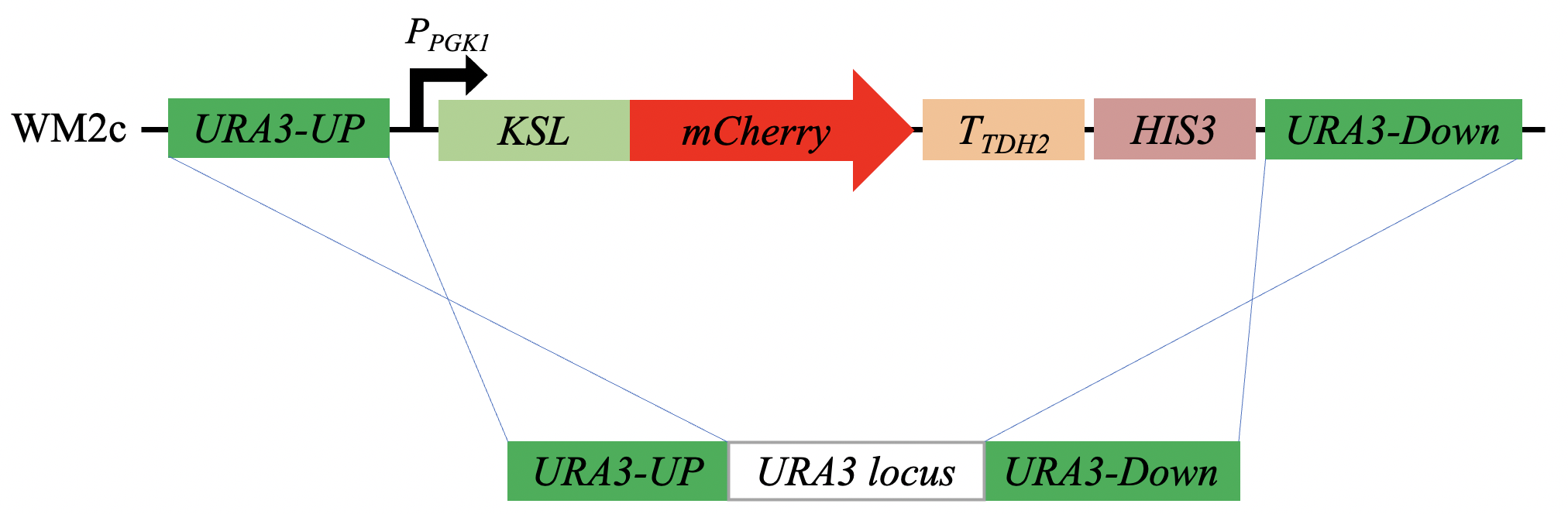

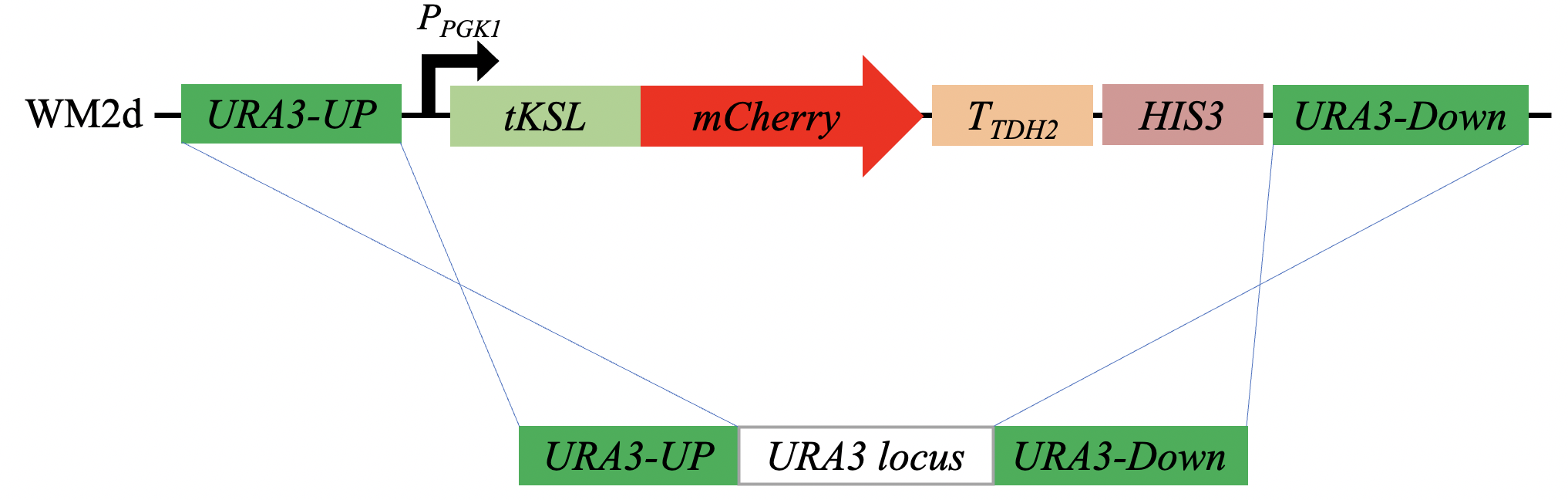

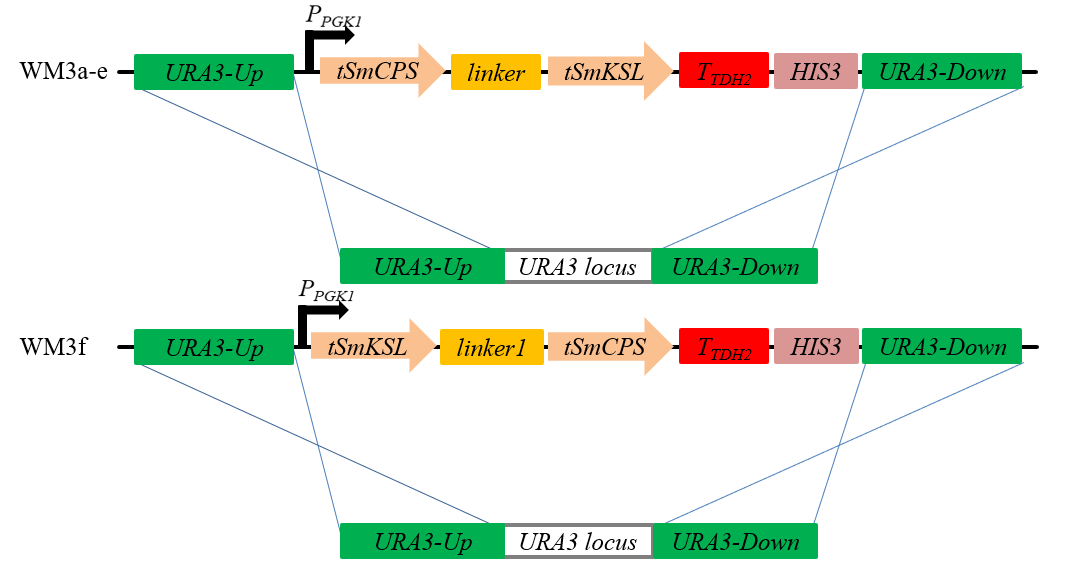

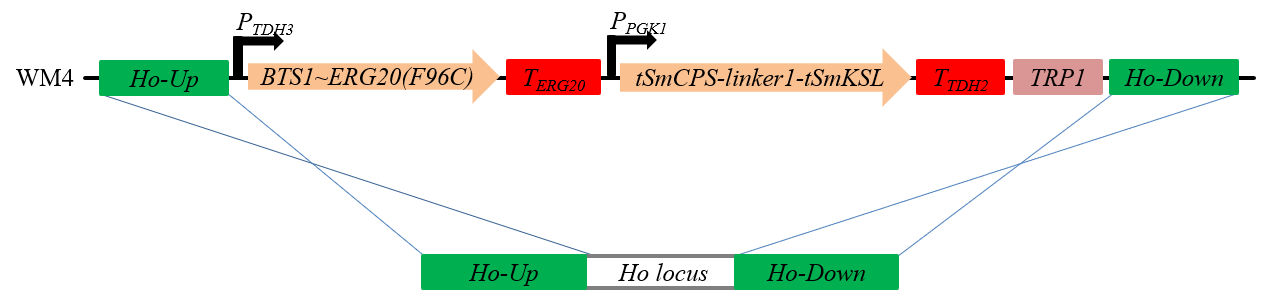

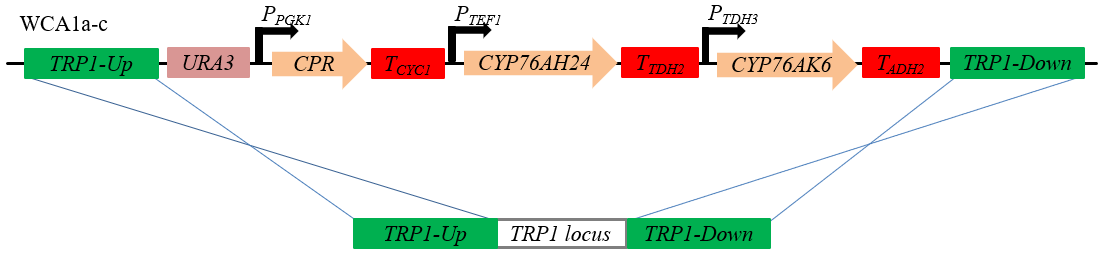

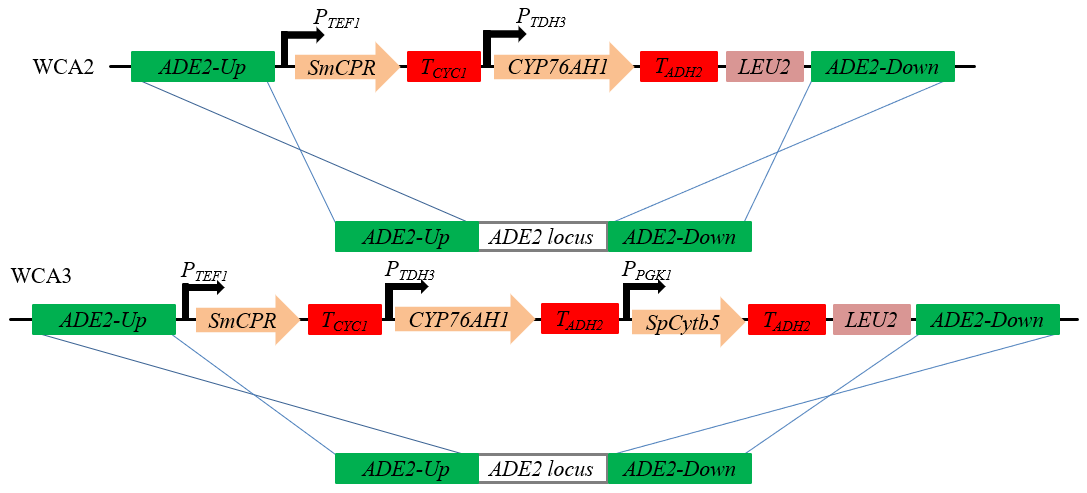

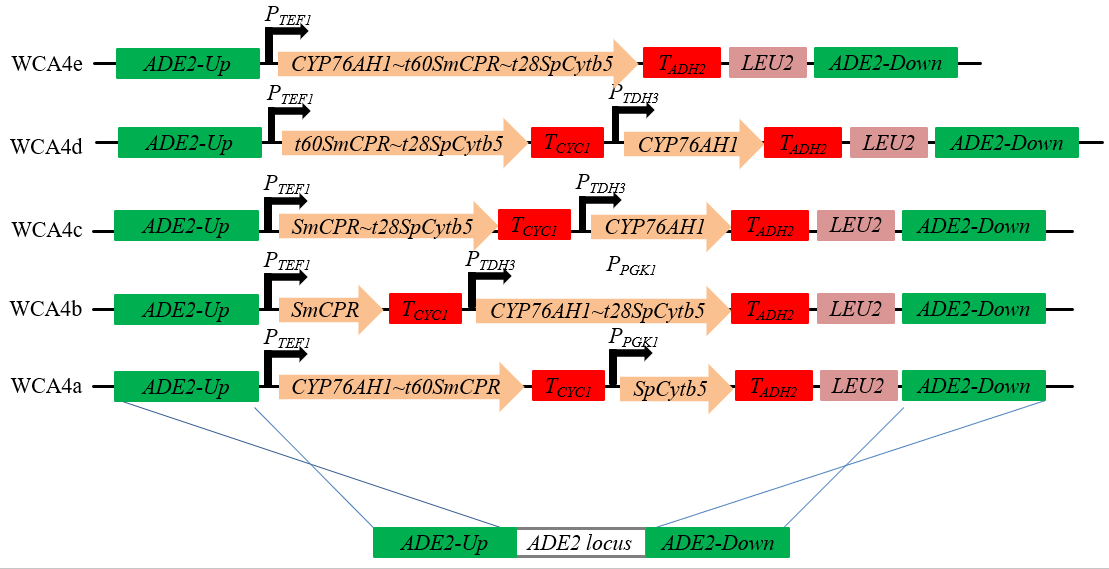


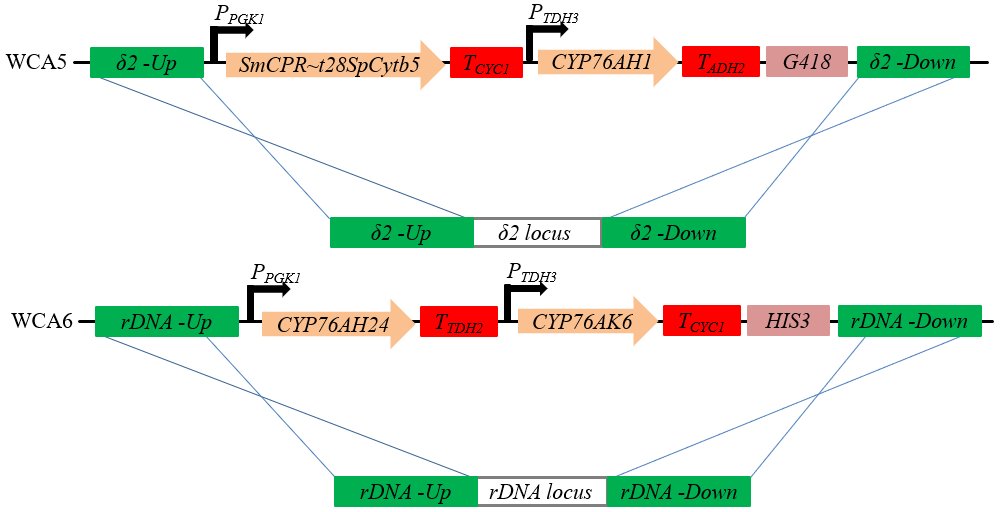

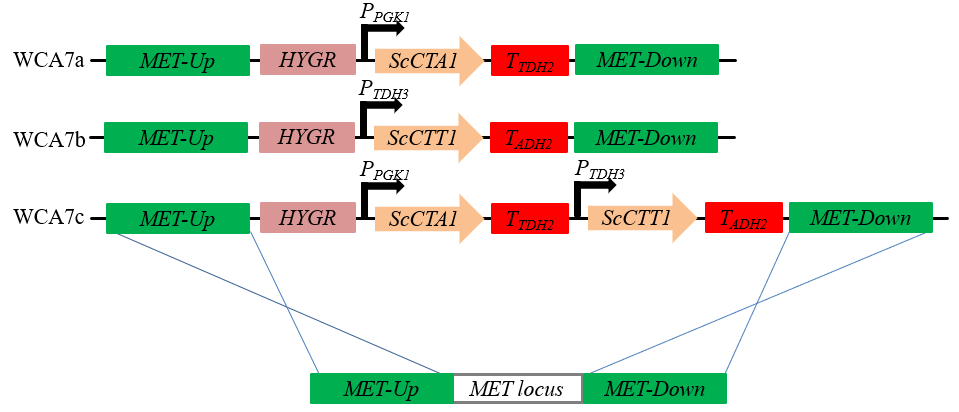
**
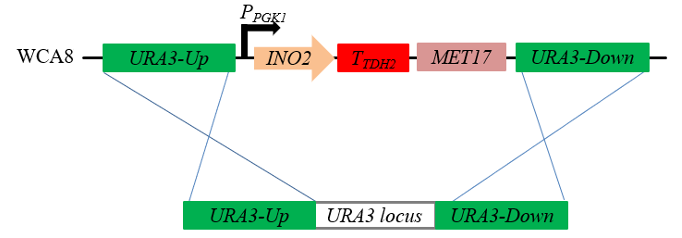
**
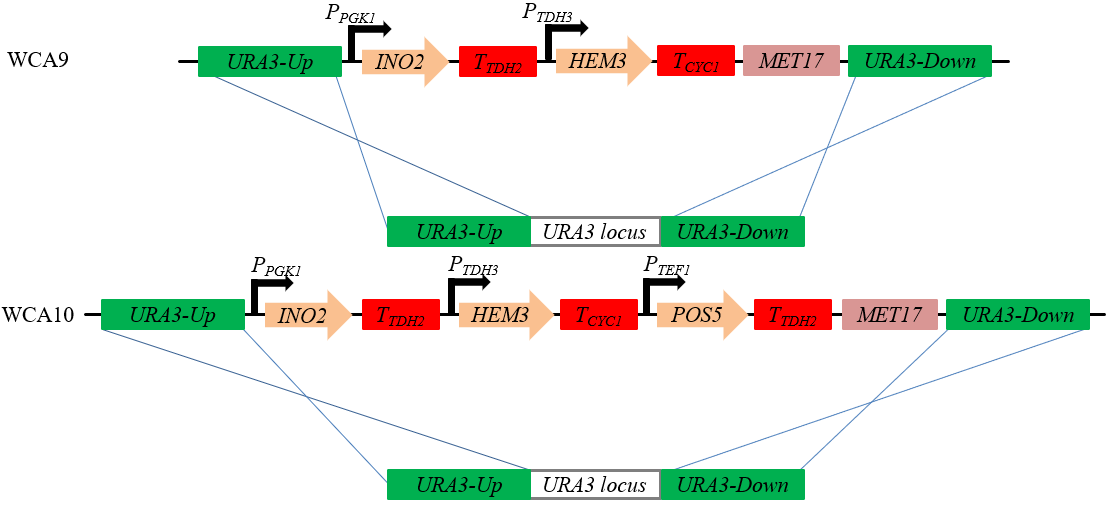

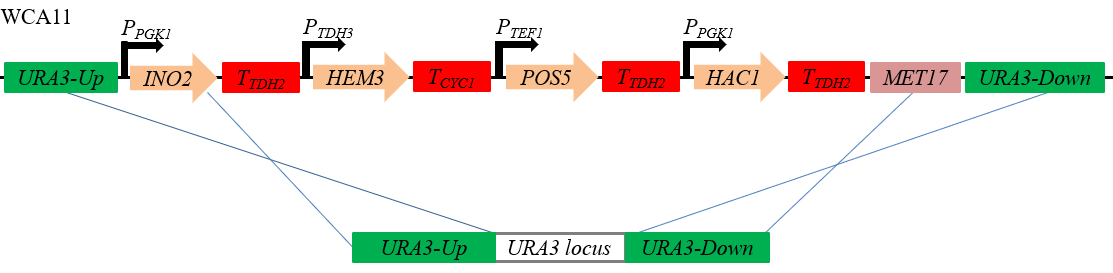


**Fig. S1** Expression cassettes construction and insertion


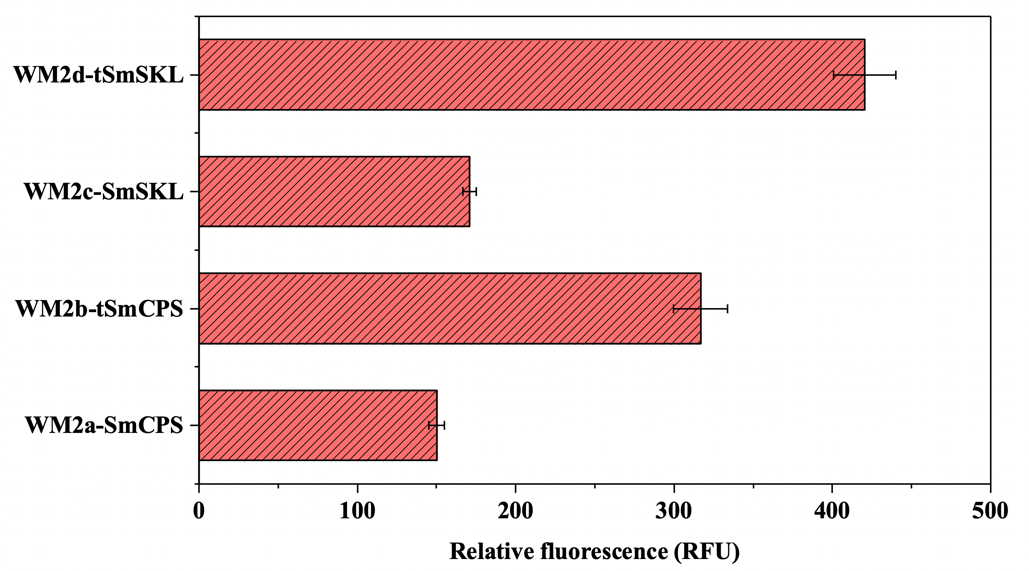


**Fig. S2** Characterization of the active expression of SmCPS, SmtCPS, SmSKL, and SmtSKL. The relative fluorescence units (RFU) were normalized to the OD_600_.


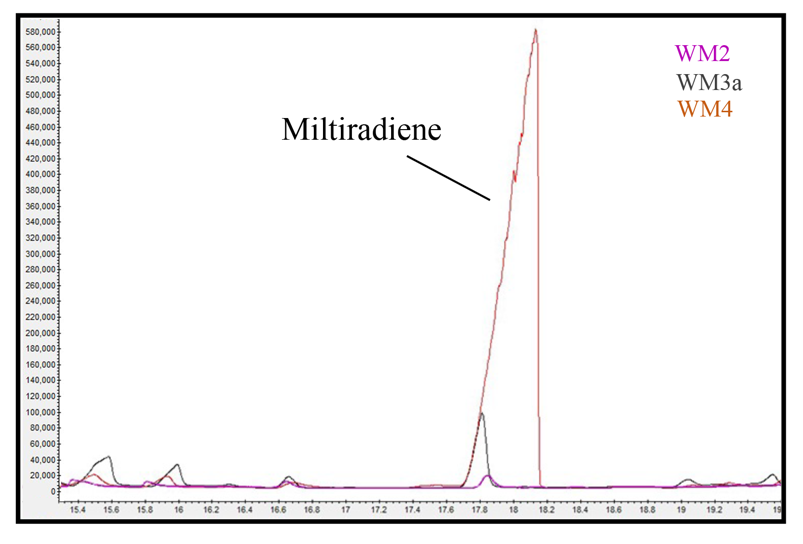


**Fig. S3** The GC-MS chromatogram for miltiradiene production by strain WM2, WM3a and WM4.


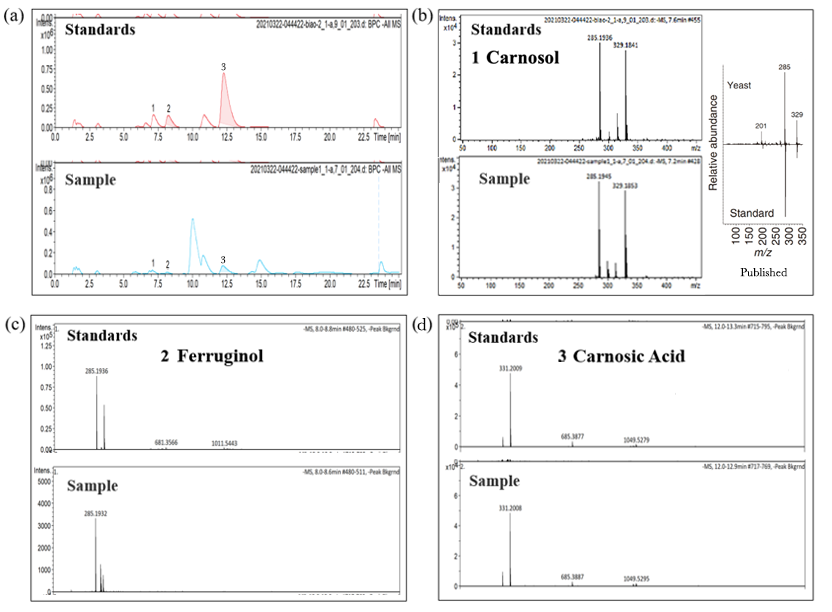


**Fig. S4** LC/MS analysis of ferruginol and CA. (a) The HPLC analysis of ferruginol and CA standard and the cell extracts from the strain WCA1c. (b) The mass spectra of sample from WCA1c and ferruginol and CA standard (selected m/z signals in the negative mode: 285, 329). The published mass spectra of carnosol (selected m/z signals in the negative mode: 285, 329) (Scheler et al., 2016). (c) The mass spectra of ferruginol standard and sample from WCA1c (selected m/z signals in the negative mode: 285). (d) The mass spectra of CA standard and sample from WCA1c(selected m/z signals in the negative mode: 331).





**Fig. S5** Copy numbers of genes integrated into the multiple-copy sites.

**
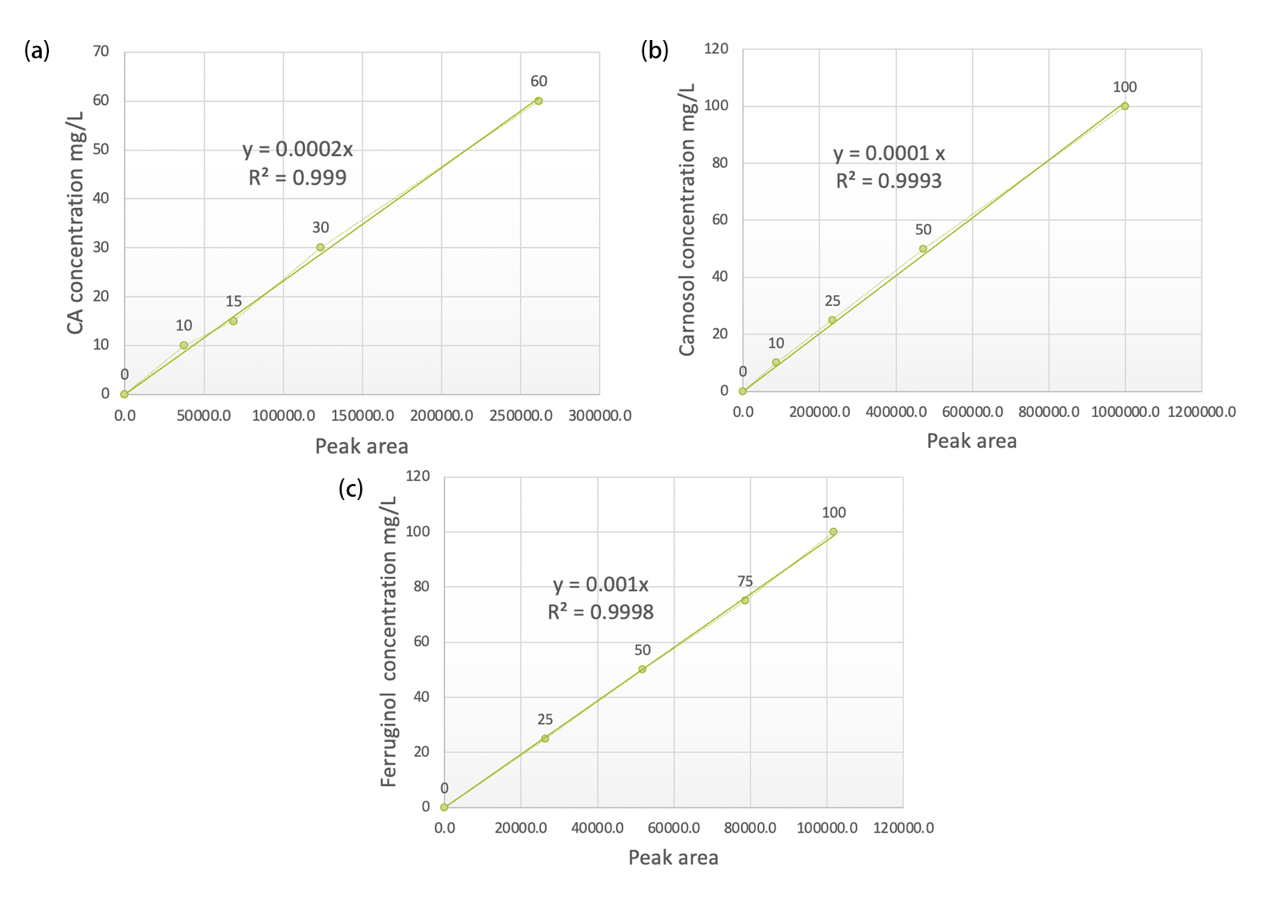
**

**Fig. S6** Calibration curve of compounds. (a) The calibration curve of CA. (b) The calibration curve of ferruginol. (c) The calibration curve of carnosol.

**
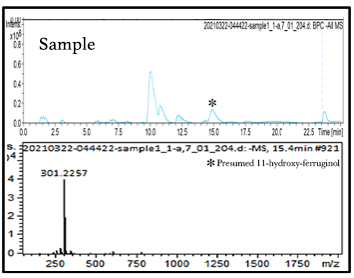
**

**Fig. S7** Presumed peak of 11-hydroxy-ferruginol. The HPLC analysis of the cell extracts from the strain WCA1c and the mass spectra of presumed 11-hydroxy-ferruginol, which was similar to the published mass spectrum of 11-hydroxyl ferruginol (LC-MS) (Scheler et al., 2016).

**References**

Scheler, U., Brandt, W., Porzel, A., Rothe, K., Manzano, D., Božić, D., et al. (2016). Elucidation of the biosynthesis of carnosic acid and its reconstitution in yeast. *Nat. Commun.* 7, 12942. doi: 10.1038/ncomms12942

Zhang, C., Li, M., Zhao, G.R., and Lu, W. (2020). Harnessing yeast peroxisomes and cytosol acetyl-CoA for sesquiterpene alpha-humulene production. *J Agric Food Chem.* 68, 1382-1389. doi: 10.1021/acs.jafc.9b07290
